# Excess of Yra1 RNA-Binding Factor Causes Transcription-Dependent Genome Instability, Replication Impairment and Telomere Shortening

**DOI:** 10.1371/journal.pgen.1005966

**Published:** 2016-04-01

**Authors:** Sandra Gavaldá, José M. Santos-Pereira, María L. García-Rubio, Rosa Luna, Andrés Aguilera

**Affiliations:** Centro Andaluz de Biología Molecular y Medicina Regenerativa CABIMER, Universidad de Sevilla, Seville, Spain; Tel Aviv University, ISRAEL

## Abstract

Yra1 is an essential nuclear factor of the evolutionarily conserved family of hnRNP-like export factors that when overexpressed impairs mRNA export and cell growth. To investigate further the relevance of proper Yra1 stoichiometry in the cell, we overexpressed Yra1 by transforming yeast cells with *YRA1* intron-less constructs and analyzed its effect on gene expression and genome integrity. We found that *YRA1* overexpression induces DNA damage and leads to a transcription-associated hyperrecombination phenotype that is mediated by RNA:DNA hybrids. In addition, it confers a genome-wide replication retardation as seen by reduced BrdU incorporation and accumulation of the Rrm3 helicase. In addition, *YRA1* overexpression causes a cell senescence-like phenotype and telomere shortening. ChIP-chip analysis shows that overexpressed Yra1 is loaded to transcribed chromatin along the genome and to Y’ telomeric regions, where Rrm3 is also accumulated, suggesting an impairment of telomere replication. Our work not only demonstrates that a proper stoichiometry of the Yra1 mRNA binding and export factor is required to maintain genome integrity and telomere homeostasis, but suggests that the cellular imbalance between transcribed RNA and specific RNA-binding factors may become a major cause of genome instability mediated by co-transcriptional replication impairment.

## Author Summary

Yra1 is an essential nuclear RNA-binding protein that plays a role in mRNA export in *Saccharomyces cerevisiae*. The cellular levels of Yra1 are tightly auto-regulated by splicing of an unusual intron in its pre-mRNA, removal of which causes Yra1 overexpression that results in a dominant-negative growth defect and mRNA export defect. We wondered whether or not *YRA1* overexpression has an effect on genome integrity that could explain the loss of cell viability. Our analyses reveal that *YRA1* overexpression causes DNA damage, confers a hyperrecombination phenotype that depends on transcription and that is mediated by RNA:DNA hybrids. YRA1 overexpression also leads to a cell senescence-like phenotype and telomere shortening. We show by ChIP-chip analysis that Yra1 binds to active chromatin and Y’ telomeric regions when it is overexpressed, in agreement with a possible role of this mRNP factor in the maintenance of telomere integrity. Our data indicate that *YRA1* overexpression correlates with replication impairment as inferred by the reduction of BrdU incorporation and the increase of Rrm3 recruitment, a helicase involved in replication fork progression, at transcribed genes and Y’ regions. We conclude that the stoichiometry of specific RNA-binding factors such as Yra1 at telomeres is critical for genome integrity and for preventing transcription-replication conflicts.

## Introduction

Messenger RNA (mRNA) is coated by RNA-binding proteins (RBPs) forming large messenger ribonucleoprotein particles (mRNPs). Many mRNA processing factors that participate in 5′-end capping, splicing, 3′-end processing, and polyadenylation are loaded co-transcriptionally to the pre-mRNA through interactions with the carboxy-terminal domain (CTD) of the RNA polymerase II (RNAPII) [1,2]. Co-transcriptional RBP loading is required for efficient transcription and RNA processing and export, and contributes to the translation process and mRNA half-life (reviewed in [3];[4,5]). Cells possess surveillance mechanisms linked to different steps of mRNP biogenesis as a way to co ntrol the quality of mRNP and the overall expression process [6]. A particularly critical feature of co-transcriptional mRNP assembly is its effect on transcription and mRNA export. Nascent transcripts are packaged by different adaptor proteins that allow the mRNP to bind the export receptor Mex67/NXF1, as termed in yeast/vertebrates, resulting in an export-competent mRNP that is transported through the nuclear pore complex (NPC) to the cytoplasm [7]; [8,9]. In yeast, Mex67 and its adaptors Yra1, Nab2 and Npl3, are recruited during transcription through specific interactions with the transcription machinery [10,11,12,13]. According to the gene-gating hypothesis [14] some transcription and mRNA export factors interact with components of the NPCs promoting the attachment of transcribed genes to the periphery, which in turn facilitate mRNA export [15] [16]. The coordination between the different mRNP biogenesis steps allows an efficient gene expression and a rapid cell response to any stimulus.

Co-transcriptional mRNP biogenesis has also been shown to be necessary for the maintenance of genome integrity. The correct formation of an mRNP particle has been proposed to prevent nascent pre-mRNA molecules from physical entanglement with DNA during transcription and the formation of R loops that would hinder transcription elongation and constitutes a block for replication fork progression [17]. One of the best-characterized examples relating mRNP biogenesis with genetic instability is provided by the THO complex, which functions at the interface transcription-mRNA export. Mutations in THO lead to a transcription-associated hyper-recombination phenotype that is R-loop dependent as shown by its partial suppression by overexpression of RNase H, an enzyme that degrades the RNA strand of DNA:RNA hybrids [18]; [19] [20]. Several studies in yeast and human cells have revealed that genome instability generated by the absence of another RNA processing factors is also R-loop-dependent [21] [22] [23] [24] [25] [26].

Yra1 is an essential nuclear RNA-binding protein that belongs to the evolutionarily conserved REF family of hnRNP-like export factors [27]. It contains an RNA-binding domain in the middle part of the protein (RBD/RRM) and two highly conserved sequences at their N- and C-termini referred as the REF-N and REF-C [28]; [27]. The REF motifs are necessary to interact with RNA [27] and the RBD/RRM domain is involved in both RNA and RNAPII CTD binding [29]. Yeast Yra1 and its metazoan counterpart, ALY/REF, play a role as an mRNA export-adaptor. It interacts with the RNA, the Sub2/UAP56 RNA-dependent ATPase and the THO complex, as well as with other mRNP factors, mediating the association of the Mex67/NXF1 RNA export factor with the mRNPs [12]. Yra1/ALY co-transcriptionally associates with mRNA contributing to its delivery to the nuclear pore complex. The loading of Yra1/ALY to active genes has been shown to be partially dependent on THO in yeast [30] [31], and on the cap-binding protein CBP80, the transcription factor Spt6 and the chromatin remodeling factor Iws1/Spn1 in mammals [32] [33]. Pcf11, a subunit of the yeast cleavage-polyadenylation factor CF1A, also participates in the cotranscriptional recruitment of Yra1 to the nascent mRNA, linking RNA export to 3’-end formation [34]. In this sense, it has been proposed that Yra1 controls polyadenylation site choice by competing with the assembly of functional CFIA at the nascent pre-mRNA [35]. In addition, Yra1/ALY has also been linked to the splicing machinery, as deduced from the observation that human ALY releases spliced mRNA from the nuclear speckles for export into the cytoplasm [36]. It seems, therefore, that Yra1/ALY could serve as a bridge between early mRNP biogenesis steps and mRNA export.

The cellular levels of Yra1 and other mRNA export factors are tightly regulated [37]. *YRA1* is one of the 5% of yeast genes that undergo splicing, containing an unusual intron in size and branch-point sequences, and its expression is negatively auto-regulated by splicing of its unusual intron [38] [39]. High Yra1 levels inhibit *YRA1* pre-mRNA splicing, and the *YRA1* pre-mRNA is exported and degraded via a highly regulated process that is dependent on the Edc3 de-capping activator and specific sequences of the *YRA1* intron [39] [40]. Interestingly, removal of the intron from the *YRA1* gene causes overexpression of Yra1, which results in a dominant-negative phenotype and an mRNA export defect [27] [41] [38]. Overexpression of other mRNP factors such as Sub2/UAP56 is also inhibitory to both cell growth and mRNA export [42] [43]. Importantly, expression of ALY and other related mRNP factors such as human THOC1/hHpr1, URH49, and CIP29/hTho1 is deregulated in tumor cells, suggesting a possible connection between mRNP metabolism and tumorigenesis [44,45];[46];[47]. Consequently, understanding the molecular basis of the growth inhibition caused by overexpression of specific RNA binding proteins would help decipher the regulation of mRNP biogenesis and its impact on cell homeostasis.

Here we examined the effect of *YRA1* overexpression in yeast. We show that *YRA1* overexpression causes DNA damage and a transcription-associated-hyperrecombination phenotype mediated by RNA:DNA hybrids. Interestingly, overexpression leads to a cell senescence-like phenotype and telomere shortening. Indeed, Yra1 binds to telomeres and its overexpression increases its occupancy and that of the Rrm3 DNA helicase at the Y’ telomeric regions, as determined by ChIP-chip analyses, and impairs replication, as determined by BrdU incorporation. The genome-wide occupancy of Rrm3 reveals a DNA replication impairment that can explain the genome instability phenotype observed. Our data suggest that stoichiometric amounts of Yra1 are critical for the maintenance of genome integrity, preventing the formation of aberrant co-transcriptional structures that may cause replication impairment.

## Results

### Expression of *YRA1Δi* leads to hyper-recombination

**Fig 1:**
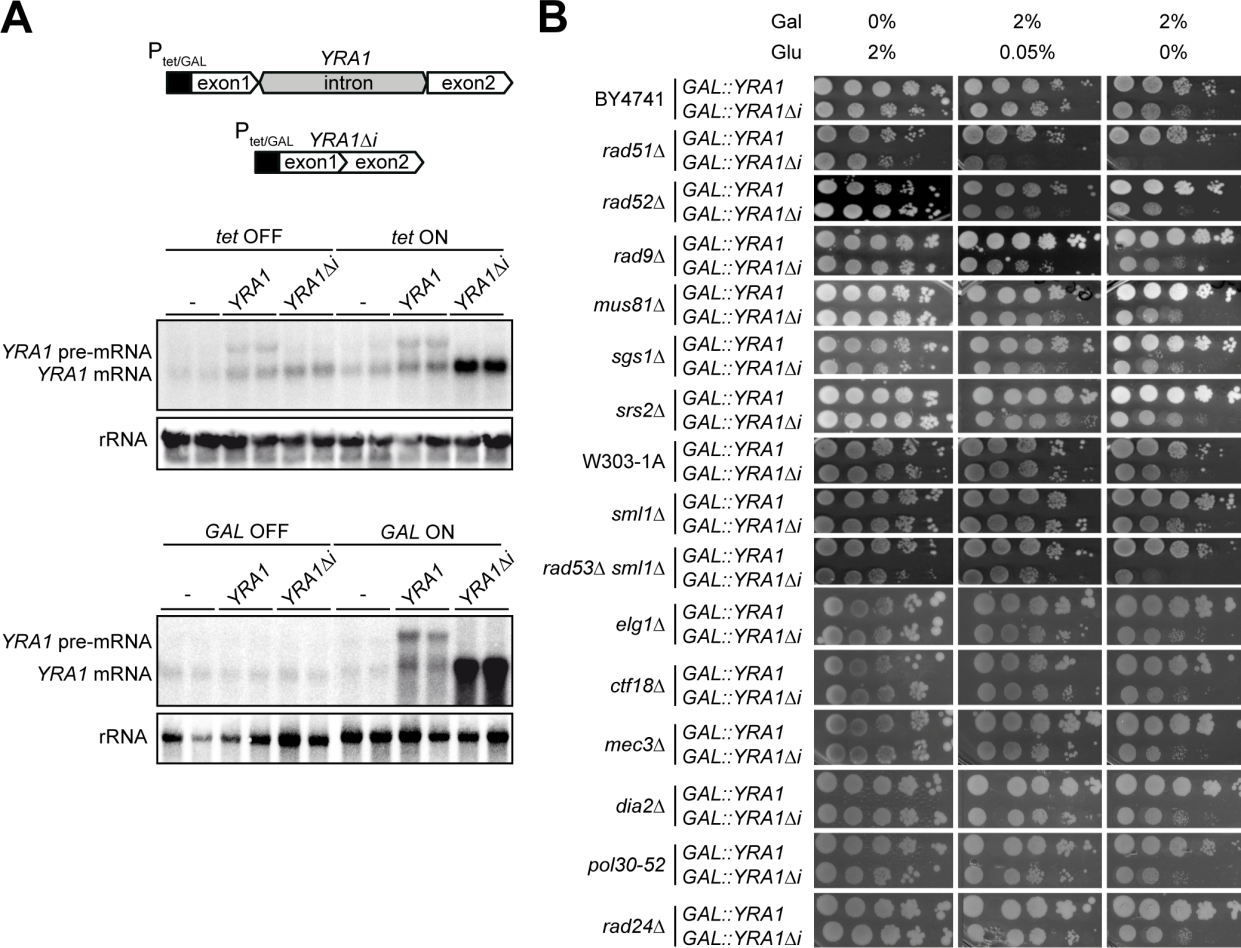
*YRA1* overexpression and cell growth inhibition. (**A**) Expression levels of the wild-type and the intron-less version of *YRA1* placed under the control of *tet* and *GAL1* promoters. Top, scheme showing the *YRA1* intron-containing and intron-less constructs is shown. Middle, northern analysis of *YRA1* mRNA in WT cells transformed with plasmids carrying *tet*::*YRA1*, *tet*::*YRA1Δi*, or the empty vector (-) (pCM184) grown in medium with 2% glucose and 5mg/ml doxycycline (*tet* OFF) and in medium with glucose and without doxycycline (*tet* ON) (upper panel). Bottom, northern analysis of *YRA1* mRNA in WT cells transformed with plasmids carrying *GAL*::*YRA1* and *GAL*::*YRA1Δi* constructs, or the corresponding empty vector (pRS413GAL) grown in medium with 2% glucose (*GAL* OFF) or in medium with 2% galactose (*GAL* ON) (lower panel). (**B**) Effect of *YRA1* overexpression in mutants of DNA-damage checkpoint factors, recombination and DNA repair proteins and factors involved in DNA metabolism. Ten-fold serial dilutions of WT (BY4741 and W303-1A) and mutant strains transformed with plasmids carrying *GAL*::*YRA1* and *GAL*::*YRA1Δi* constructs, and plated on minimal selective medium with either glucose (Glu) or galactose (Gal) are shown. Photographs were taken after 3 days of growth at 30°C.

Given the interaction of Yra1 with the THO complex, and the role of THO at the interface of transcription and genetic instability [30] [48], we first wondered if overexpression of *YRA1* could interfere with these functions. For this purpose, we cloned the *YRA1* gene (*YRA1*) and a cDNA copy of *YRA1* (*YRA1Δi*) under the control of the Tet-off promoter, which is repressed upon doxycycline addition, and under the strong *GAL1* promoter. *YRA1* overexpression is achieved when expression is driven from *YRA1Δi* constructs, in agreement with previous reports (Fig 1A) [41] [38]. In *tet*:*YRA1Δi* transformants an increase in *YRA1* mRNA levels could be observed in medium without doxycycline (ON condition) and this effect was exacerbated in *GAL*::*YRA1Δi* cells grown in galactose-containing medium. To gain insight into the molecular basis of the growth inhibition phenotype associated with *YRA1* over-expression we investigated whether this effect was increased in the absence of factors involved in the maintenance of genome integrity such as recombination factors, DNA-damage checkpoint proteins, DNA helicases and other factors involved in DNA metabolism (Fig 1B). The selected mutants were transformed with *GAL*::*YRAΔi* and *GAL*::*YRA1* constructs and cultured in 2% galactose-containing medium, with and without the presence of small amounts of glucose (Fig 1B). Interestingly, a strong growth defect was observed in checkpoint (*RAD53*) and recombination (*RAD51*, *RAD52)* mutants when *YRA1Δi* was expressed in 2% galactose-containing medium (Fig 1B). Growth of *rad51Δ* mutants was severely affected even in the presence of small amounts of glucose. *YRA1* overexpression also causes a little growth inhibition in checkpoint and DNA helicase mutants such as *rad9Δ* and *sgs1Δ*, but no effect was observed in other checkpoint and DNA helicase mutants such as *mec3Δ* and *srs2Δ*. We did not detect growth impairment in mutants of factors involved in DNA metabolism at the conditions assayed. The results suggest that *YRA1* overexpression could have a specific impact on genome integrity, leading to DNA breaks that demand the DNA damage checkpoint and recombination machineries to prevent its negative effect on cell proliferation.

**Fig 2:**
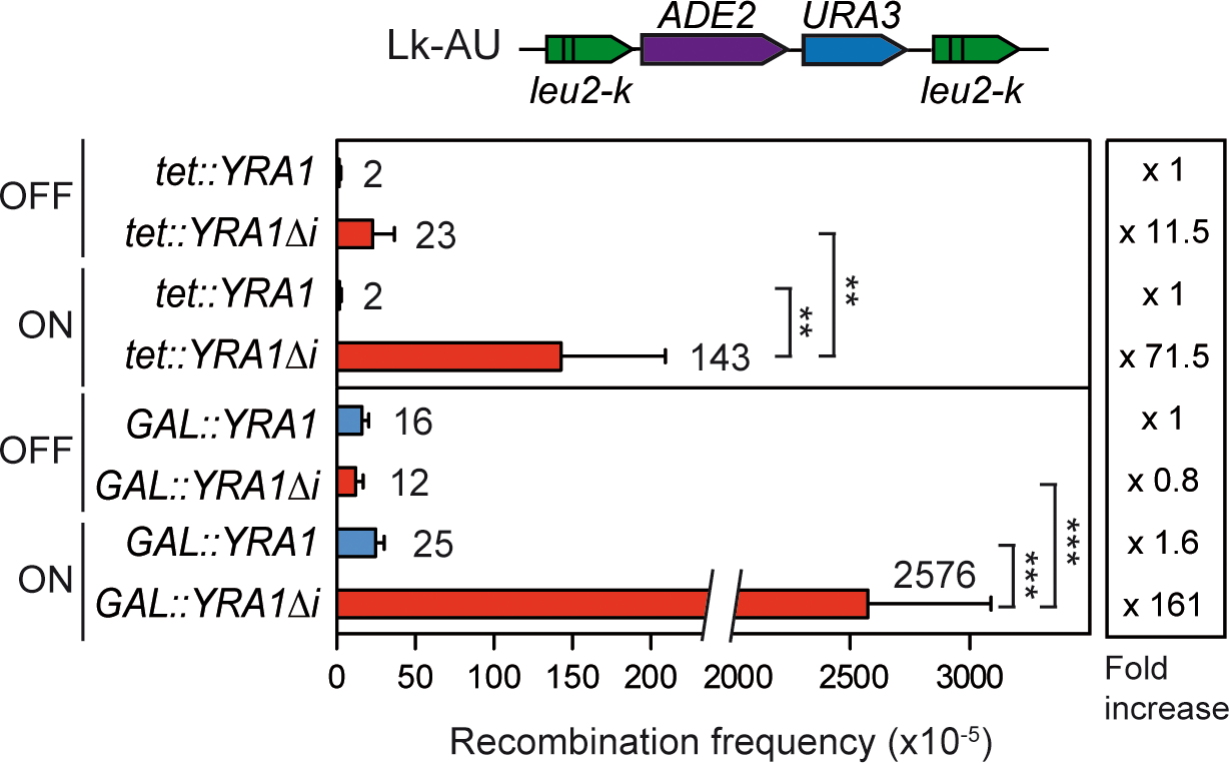
Genome instability in *YRA1*-overexpressing cells. (Recombination frequencies of the WT strain carrying the chromosomal *leu2-k*::*ADE2-URA3*::*leu2-k* system and transformed with either *tet*::*YRA1*, *tet*::*YRA1Δi*, *GAL*::*YRA1* or *GAL*::*YRA1Δi* constructs are shown. Average and SD of four fluctuation tests made with six independent colonies each one are shown. The asterisks indicate statistically significant differences, according to Student's t-tests (*, Ρ< 0.05; **, Ρ< 0.005; ***, Ρ< 0.0005).

Next, we investigated the effect of *YRA1* overexpression on genome integrity by assaying spontaneous recombination levels. Using the chromosomal direct-repeat recombination system *leu2-k*::*ADE2-URA3*::*leu2-k*, we observed a 71.5—and 161-fold increase in recombination caused by *YRA1* overexpression from the plasmid-borne systems *tet*::*YRA1Δi* and *GAL*::*YRA1Δi*, respectively (Fig 2). No significant increase in recombination was observed after expression of *YRA1* intron-containing constructs *tet*::*YRA1* and *GAL*::*YRA1*. These data indicate that *YRA1* overexpression confers a hyper-recombination phenotype.

Finally, since overexpression of Sub2, which forms a heterodimer with Yra1, is also inhibitory to both cell growth and mRNA export [42] [43], we wondered whether imbalance of these factors could be the cause of the cell growth inhibition. Since it has been shown that Sub2 over-abundance impairs mRNA export and reduces Yra1 recruitment to genes [49], we assayed whether simultaneous overexpression of *YRA1* and *SUB2* could restore the wild-type phenotype. Interestingly, growth inhibition was slightly suppressed by co-overexpression of both proteins (S1A Fig). However, the hyperrecombination phenotype conferred by multicopy *YRA1* was not suppressed by *SUB2* overexpression, suggesting that the effect of *YRA1* overexpression is Sub2-independent (S1B Fig).

### Hyper-recombination caused by *YRA1* overexpression is transcription-dependent

**Fig 3:**
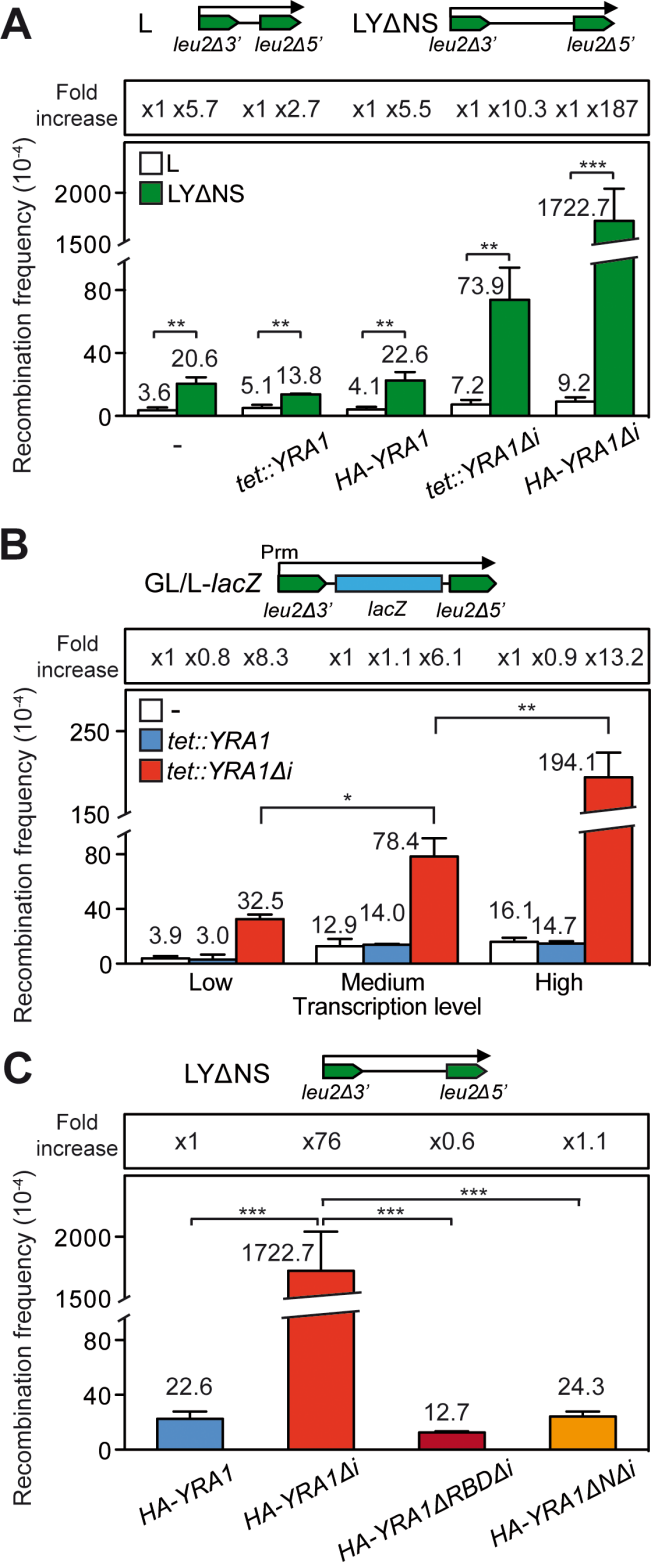
Hyper-recombination in *YRA1*-overexpressing cells. Effect of transcription on the hyper-recombination phenotype conferred by *YRA1* overexpression. (**A**) Recombination analysis of the plasmid-borne recombination systems L and LYΔNS in WT cells transformed with either *tet*:*YRA1*, *tet*:*YRA1Δi*, HA-*YRA1 and HA-YRAΔi* constructs. (**B**) Recombination frequency in WT cells transformed with *tet*:*YRA1* or *tet*:*YRA1Δi* constructs using the plasmid-borne direct-repeat GL-*lacZ* system expressed under the control of the *GAL* promoter in glucose or galactose (low and high transcription levels, respectively) and the L-*lacZ* system expressed under the control of the *LEU2* promoter (medium transcription level). Gray boxes represent *LEU2* repeats. Arrows indicate the transcript produced. Prm, Promoter. (**C**) Recombination analyses in cells overexpressing truncated Yra1 proteins. Recombination frequencies were determined using the plasmidic recombination system LYΔNS in cells transformed with constructs expressing the *YRA1* gene (*HA-YRA1*), the *YRA1* cDNA (*HA-YRA1Δi*), or truncated *YRA1* cDNA versions (*HA*-*YRA1ΔRBDΔi* or *HA*-*YRA1ΔNΔi*) lacking either the RNA binding domain, or the N-terminal conserved sequence REF-N. In all these constructs *YRA1* expression is under the control of the *YRA1* promoter, and the resultant Yra1 protein is hemaglutinin (HA)-tagged. The average median value and SD of 3–4 fluctuation tests are shown. Asterisks indicate statistically significant differences between the strains indicated, according to Student's t-tests (*, Ρ< 0.05; **, Ρ< 0.005; ***, Ρ< 0.0005).

Next we investigated whether the hyper-recombination phenotype caused by *YRA1* overexpression was associated with transcription. For this we measured the recombination frequencies in the L and LYΔNS direct-repeat recombination systems (Fig 3A). These systems are based on the same direct-repeats (600-bp internal fragments of the *LEU2* gene sharing 300-bp of homology) that are transcribed from the *LEU2* promoter, but they differ in the length of the transcribed intervening sequence (31 bp for L, and 3.7 kb for LYΔNS) [50]. We found that the strong hyper-recombination phenotype was only observed with *YRA1* overexpression (*tet*::*YRA1Δi* and *HA-YRA1Δi*) when the long 3.7kb sequence was transcribed (LYΔNS system) (Fig 3A).

The result suggests that *YRA1* overexpression confers a transcription-dependent genetic instability phenotype since the longer the transcribed region is the higher the increase in recombination. To confirm this, we determined the effect of *YRA1* overexpression in the L-*lacZ* and GL-*lacZ* recombination systems that contain the GC-rich sequence *lacZ* between 0.6-kb *leu2* direct-repeats transcribed from different promoters, *LEU2* promoter (L-*lacZ* system) or *GAL1* promoter (GL-*lacZ* system) (Fig 3B). We have previously used these systems to report the transcription-associated recombination (TAR) phenotype of different mRNP biogenesis mutants [51]; [52]. Recombination analyses were carried out in wild-type cells transformed with plasmids carrying the *tet*::*YRA1* or *tet*::*YRA1Δi* constructs or with the empty vector. Transformants were grown in the absence of doxycycline to allow *YRA1* expression (ON conditions), and under conditions of low (*GAL1* promoter in 2% glucose), medium (*LEU2* promoter in 2% glucose) and high levels of transcription (*GAL1* promoter in 2% galactose) of the recombination system used in each case. As can be seen in Fig 3B, the recombination frequencies of *tet*::*YRA1* expressing cells were similar to those of wild-type cells transformed with the empty plasmid. In contrast, in the case of *tet*::*YRA1Δi* cells, the higher the strength of transcription of the recombination system used, the stronger the increase in recombination. Altogether, these data indicate a statistically significant increase in recombination levels in *YRA1* overexpressing cells with respect to the wild type that is transcription-dependent.

Next, we studied the relevance of the conserved RBD/RRM and REF domains of the Yra1 protein in the genome instability phenotype. We determined the recombination frequency of the LYΔNS system in cells expressing, either the *YRA1* gene (*HA-YRA1*), the *YRA1* cDNA (*HA-YRA1Δi*), or truncated *YRA1* cDNA versions lacking either the RBD/RRM or the REF-N domains (HA-*YRA1ΔRBDΔi* or HA-*YRA1ΔNΔi*, respectively)(Fig 3C); [27]. The HA-tagged Yra1 constructs used are under the native *YRA1* promoter instead of the *GAL/tet* promoters, and show protein levels expected for the conditions used. Cells carrying the *HA-YRA1Δi* construct showed higher levels of tagged Yra1 than those with *HA-YRA1* (S2 Fig). However, in the case of cells transformed with HA-*YRA1ΔRBDΔi* the levels of proteins were very low as detected by western blot with anti-HA antibody, in agreement with previous published data [27]. The recombination levels of cells overexpressing truncated Yra1 proteins were close to those of cells not overexpressing *YRA1* (*HA*-*YRA1*), in contrast to the 76-fold increase in recombination shown by cells overexpressing the full Yra1 protein (*HA*-*YRA1Δi*) (Fig 3C). Since the amount of HA-*YRA1ΔRBD* protein detected is too low, further experiments are needed to delineate which domains and properties of Yra1 are the most relevant for genome instability when the protein is overexpressed.

### The genome-instability phenotype conferred by *YRA1* overexpression is mediated by RNA:DNA hybrids

**Fig 4:**
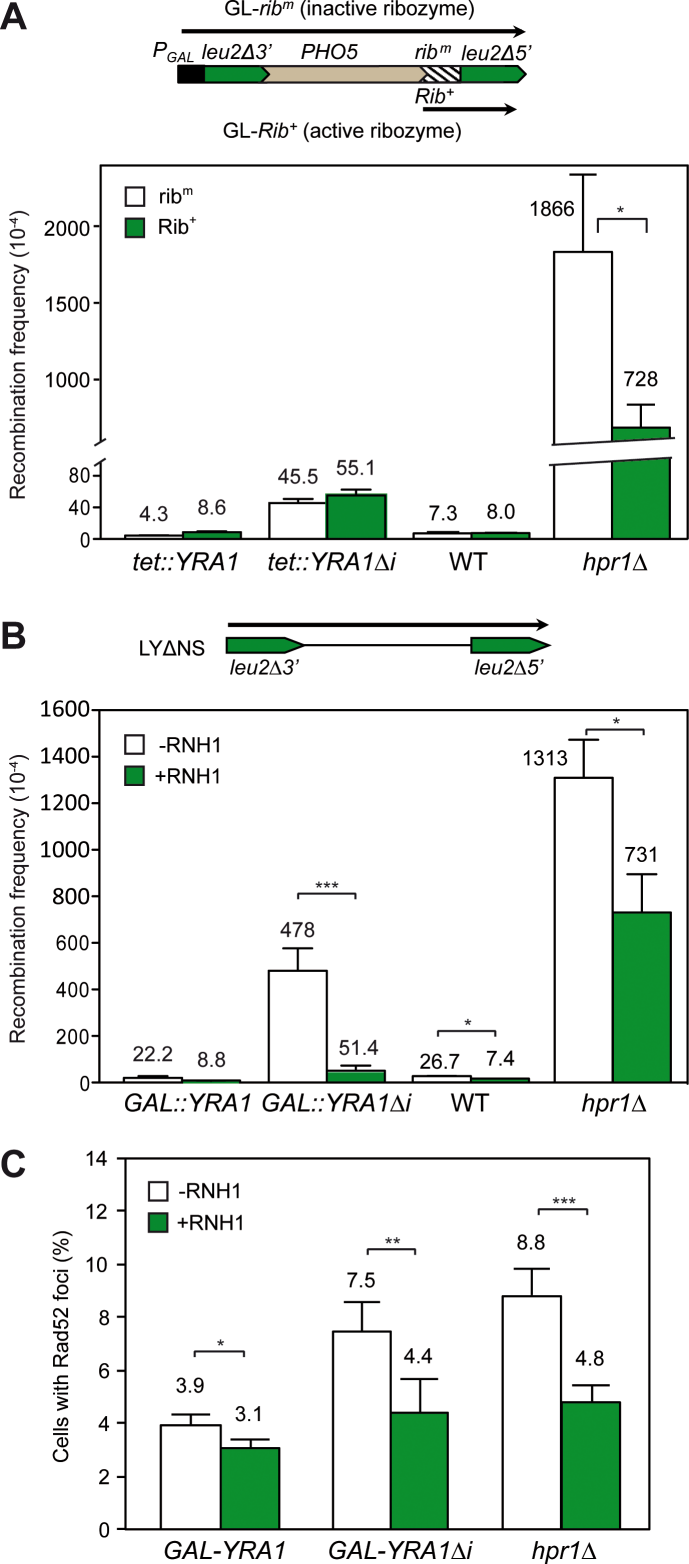
R loop-dependency of hyperrecombination in *YRA1*-overexpressing cells (**A**) Recombination frequencies in WT cells transformed with *tet*:*YRA1* or *tet*:*YRA1Δi* constructs using recombination systems GL-*Rib*^*+*^ and GL-*rib*^*m*^. The *hpr1Δ* mutant was used as positive control. Direct-repeat recombination systems GL-*Rib*^*+*^ and GL-*rib*^*m*^ contain the *PHO5-Rib*^*+*^ or *PHO5- rib*^*m*^ sequences flanked by two truncated copies of *LEU2* in direct orientation under the *GAL1* promoter. These systems contain an active or inactive 52-bp ribozyme (*Rib*), respectively. The *rib*^*m*^ system (inactive ribozyme) yields a long transcript, whereas in the *Rib*^*+*^ system (active ribozyme) self-cleavage of the *PHO5-Rib* transcript leads to a shorter mRNA (represented by arrows). Experiments were performed in 2% galactose to allow expression of the direct repeats. (**B**) Effect of RNH1 overexpression on the recombination frequency of the LYΔNS system in cells carrying *GAL*::*YRA1 or GAL*::*YRA1Δi* constructs and transformed with either the pGAL-RNH1 or the empty vector. Experiments were performed in galactose to achieve Yra1 and Rnh1 overexpression. The *hpr1Δ* mutant was included as positive control. The average median values and SD of 3–4 fluctuation tests are shown. (**C)** Effect of RNH1 overexpression on Rad52 foci formation in cells carrying *GAL*::*YRA1* or *GAL*::*YRA1Δi* constructs and transformed with either the pGAL-RNH1 or the empty vector. The *hpr1Δ* mutant was included as positive control. Asterisks indicate statistically significant differences between the strains indicated, according to Student's t-tests (*, Ρ< 0.05; **, Ρ< 0.005, ***, Ρ< 0.0005).

Since *YRA1* overexpression confers an increase in transcription-associated recombination (TAR), we wondered whether this was dependent on the nascent mRNA and whether was mediated by R loops. We first used the GL-*Rib*^*+*^ and GL-*rib*^*m*^ repeat recombination systems that contains an active (Rib^+^) and inactive (rib^m^) hammerhead ribozyme, respectively [18]. Both the Rib^+^ and rib^m^ constructs synthesize a long mRNA, but upon transcription the active hammerhead ribozyme cleaves the nascent transcript shortening the mRNA fragment that remains attached to RNAPII. Fig 4A shows that the *tet*::*YRA1Δi* construct leads to a 10-fold increase in recombination with respect to the *tet*::*YRA1* construct, but the recombination frequencies were the same in the GL-*Rib*^*+*^ and in the GL-*rib*^*m*^ systems, in contrast to the control *hpr1Δ*, a THO mutant [18] in which the ribozyme-mediated cleavage of the nascent RNA partially suppresses the hyper-recombination phenotype. The data suggests that the length of the nascent RNA does not influence genome integrity in cells overexpressing Yra1. To assess the possibility that R loops could contribute to hyper-recombination in *YRA1*-overexpressing cells, we assayed whether hyper-recombination could be suppressed by overexpression of RNase H, which digests the RNA moiety of RNA:DNA hybrids (Fig 4B). For this we tested recombination in the LYΔNS system in cells expressing *GAL*::*YRA1* or *GAL*::*YRA1Δi*, transformed either with a plasmid carrying *RNH1* under the *GAL1* promoter, or with the corresponding empty vector. Overexpression of RNase H1 leads to a significant and clear reduction (9-fold) in the recombination levels of cells overexpressing *YRA1* (*GAL*::*YRA1Δi*). A partial suppression in the hyper-recombination *hpr1Δ* mutant, used as positive control, and a reduction in the basal recombination levels in wild-type cells were also observed.

We tried to see whether expression of the human cytidine deaminase AID could also be used as an indirect genetic measure of R-loop formation in *YRA1*-overexpressing cells. This enzyme acts on single stranded DNA and has been used as a tool to infer R-loop accumulation [53] by exacerbating hyper-recombination in different yeast RNA biogenesis mutants ([53]; [52] [54]). Recombination was slightly increased under *tet*::*YRA1Δi* overexpression with AID (S3 Fig) but the increase was low. Since the accessibility of AID to co-transcriptional R loops is mediated by the transcription apparatus [55], we cannot discard that an excess of Yra1 may indirectly interfere with AID accessibility.

The data suggest that Yra1 overexpression causes more recombination events and consequently more DNA breaks. Consequently we tested this prediction by quantifying the number of Rad52 foci. As can be seen in Fig 4C overexpression of *GAL*::*YRA1Δi* leads to a significant increase in the percentage of cells with Rad52 foci compared with that of the *GAL*::*YRA1* expression system in wild-type cells, indicating that *YRA1* overexpression leads to an increase in the accumulation of recombinogenic DNA breaks. To determine whether the increase in DNA breaks was dependent on R loops we assayed whether Rad52 foci accumulation were suppressed by RNase H1 overexpression. To allow sufficient time for RNH1 overexpression and action, experiments were performed from mid-log growing cells after 15 hours of inducing overexpression of both *YRA1* and *RNH1*. As can be seen in Fig 4C, the significant increase caused by *YRA1* overexpression (2-fold) was suppressed by *RNH1* overexpression. The result was similar to that of the positive R-loop-dependent hyper-recombinant control *hpr1Δ* mutant (Fig 4C). Altogether, these data indicate that R loops mediate genome instability triggered by *YRA1* overexpression.

### *YRA1* overexpression causes a senescence-like phenotype and telomere shortening

**Fig 5:**
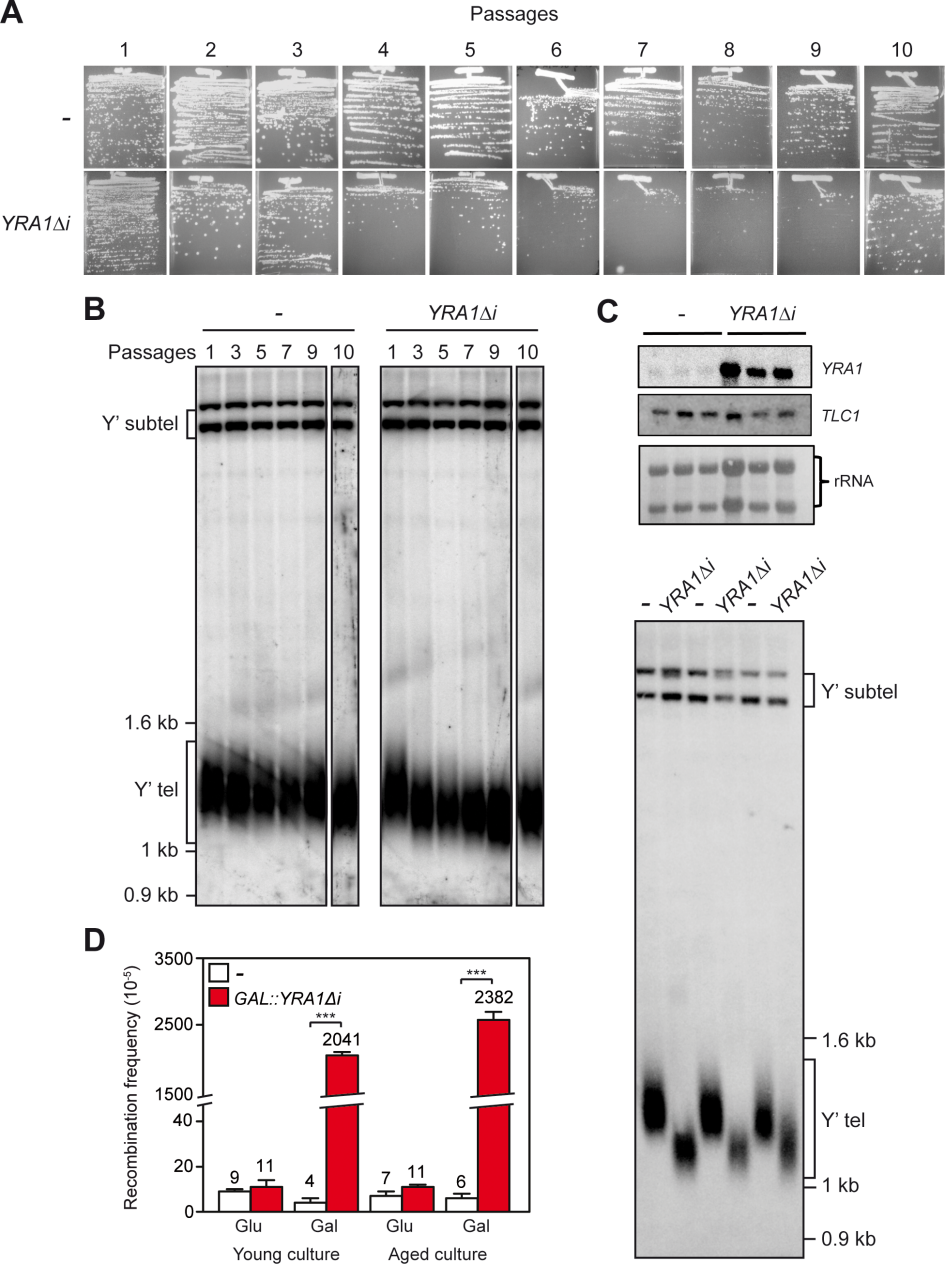
Senescence-like phenotype and telomere shortening in *YRA1*-overexpresssing cells. (**A**) Plate images of serial passaging of WT transformed with the *GAL*::*YRA1Δi* construct or the corresponding empty vector. The results of ten successive streaks-outs on medium with 2%-galactose supplemented with 0.05% glucose are shown. Here and in further figures the passage number is indicated above image. (**B**) Southern analysis of wild-type cells transformed with the *GAL*::*YRA1Δi* or the empty plasmids to study telomere length dynamic at the passages shown in A. Sub-telomeric and telomeric fragments are visualized using a telomeric-specific probe (Y’ probe). (**C**) Northern analysis of the *TLC1* gene from three different transformants carrying either the empty vector or the *GAL*::*YRA1Δi* construct, after 80 generations. mRNA levels were quantified and normalized with respect to the rRNA levels of each sample. An internal 589-bp 25S rDNA fragment obtained by PCR was used as probe. Southern analysis of genomic DNA from the three different *GAL*::*YRA1Δi* (Δi) or empty plasmid transformants (-) grown successively in liquid culture for 70–80 generations.(**D**) Recombination frequencies of WT young cultures and aged cultures carrying the chromosomal *leu2-k*::*ADE2-URA3*::*leu2-k* recombination system and either the *GAL*::*YRA1Δi* construct or the corresponding empty vector. Cultures were grown on medium with 2%-galactose supplemented with 0.05% glucose. Other details as in Fig 2.

It has been established that genome instability contributes to cell lifespan by different mechanisms, such as rDNA loss, mitochondrial dysfunction, DNA replication defects and telomere erosion [17] [56]. We noticed that yeast cells transformed with the *GAL*::*YRA1Δi* construct showed a decrease in colony formation after successive passages in galactose-containing medium. As *YRA1* overexpression inhibits cell growth, we wondered whether this dominant-negative effect could be due to a senescence-like process. Therefore we performed successive streak-outs of yeast cells transformed with the *GAL*::*YRA1Δi* construct or the corresponding empty plasmid (Fig 5A). We compared the cell viability of transformants in medium with 2%-galactose supplemented with 0.05% glucose to achieve *YRA1* medium-level overexpression and minimize the inhibitory effect caused by high overexpression. As shown in Fig 5A, the colony size of cells expressing *YRA1Δi* was progressively smaller compared with those of colonies from the first streak-outs. We observed a poor cell growth after passages 5–6 that seems to be recovered at passage 10, suggesting that *YRA1* overexpression was leading to a senescence-like phenotype.

As telomere shortening has been commonly associated with loss of cell viability and senescence [57] [58] [59], we tested whether the growth defect phenotype caused by *YRA1* overexpression was accompanied by changes in telomere length. Southern analysis with *Xho*I-digested genomic DNA from transformants was carried out to analyze telomere length dynamics at different passages. Fig 5B shows that telomeres were shortened at early passages (see passage 3) maintaining the small size after further passages. Given that Yra1 is an RNA-binding protein that plays a role in gene expression, we assayed whether the effect on telomere length was a consequence of deregulation of the RNA component of telomerase *TLC1*. Northern analysis (Fig 5C) indicates that the levels of *TLC1* RNA are similar in *YRA1Δi* overexpressing cells and control cells after 70–80 generations, whereas the telomere was reduced in cells overexpressing *YRA1* (Fig 5C).

Next, we analyzed telomere length in different *yra1* mutants to assay whether failure of specific Yra1 domains could be responsible of the observed telomere shortening. Southern analysis with genomic DNA from *yra1Δ* strains complemented either with *YRA1* gene (*YRA1*), the *yra1-1* allele [60], the *YRA1* cDNA (*YRA1Δi*), or the truncated *YRA1* cDNA lacking the RBD domain (*YRA1ΔRBDΔi*) revealed that telomere length was not significantly affected in *yra1-1* mutants, but it was shorter under *YRA1* overexpression (see YRA1*Δi* strain in S4 Fig). Since this reduction in telomere length was not observed in *YRA1ΔRBDΔi* cells, despite the lower levels of expression of this construct (S2 Fig), the data could suggest that the ability of Yra1 to bind to RNA could be necessary for the telomere shortening.

To further analyze the possible relationship between hyper-recombination associated with *YRA1* overexpression and the senescence-like phenotype, we performed recombination analyses of colonies from young (10 or less generations) and aged cultures (70–80 generations) carrying the chromosomal recombination system *leu2-k*::*ADE2-URA3*::*leu2-k* (Fig 5D). As can be seen, cells from standard young cultures show recombination frequencies similar to those of aged cells (2.0x10^-3^ versus 2.5 x10^-3^), implying that hyper-recombination is independent of aging.

### Genome-wide distribution of overexpressed Yra1 is enriched toward the 3’ end of transcribed genes and at telomeres

**Fig 6:**
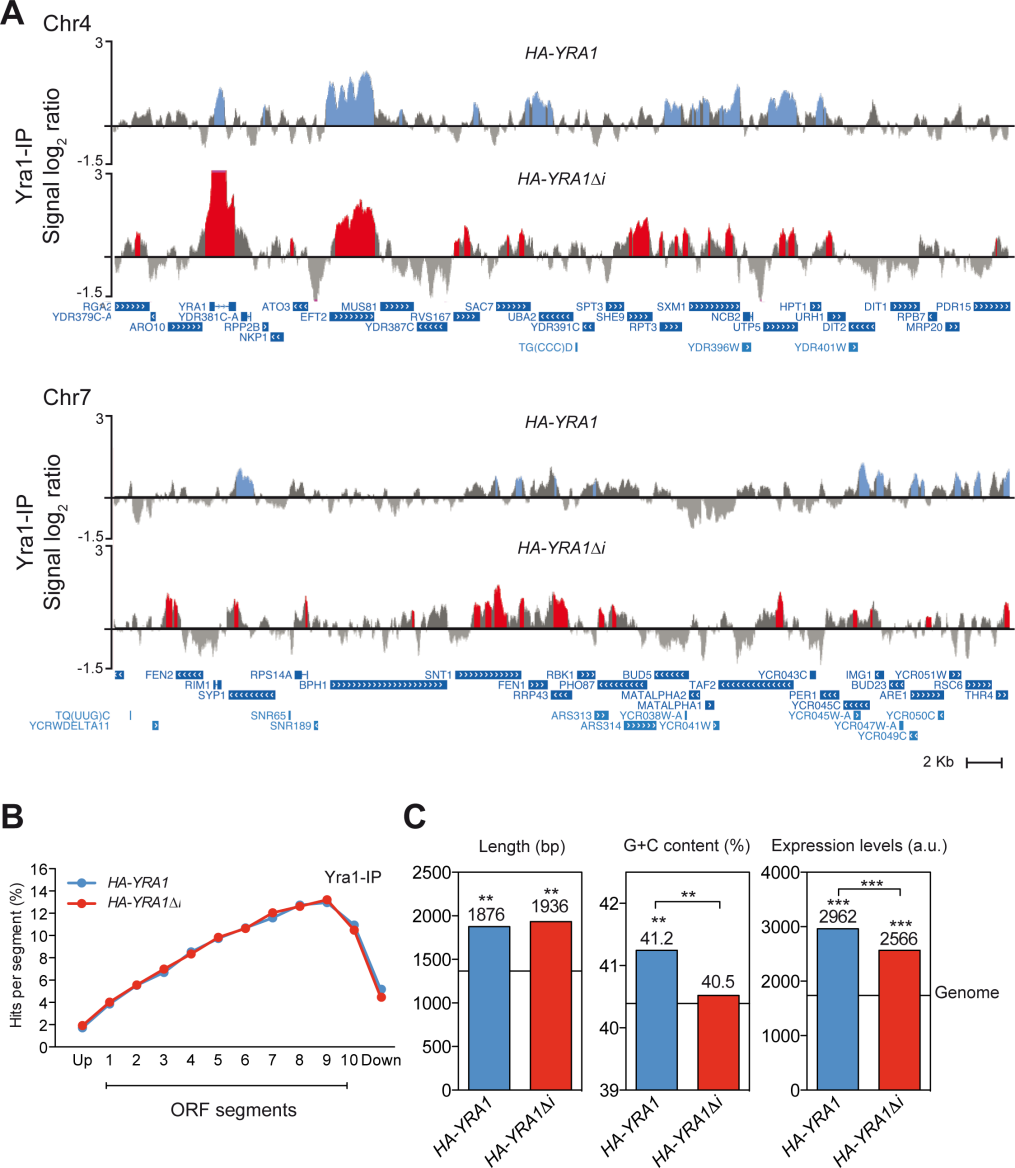
Genome-wide analyses of Yra1 recruitment under Yra1 WT (*HA-YRA1*) and Overexpression (HA-*YRA1Δi*) conditions. (**A**) Fragments of chromosome IV and Chromosome VII are plotted with the signal log_2_ ratio values. Blue (*HA-YRA1*) and Red (*HA-YRA1Δi*) histograms represent the significant Yra1 binding clusters *(P<0.01, minimum run>100pb, maximum gap < 250bp). Genes and other features are represented according to the *Saccharomyces* Genome Database (SGD). Blue bars with white arrows are indicated according to the direction of transcription. (**B**) Composite profile of Yra1 occupancy detected by ChIP-chip along the average ORF plotted as Yra1 percentage of ChIP clusters per segment in WT (*HA-YRA1*) and *YRA1* Overexpression (HA-*YRA1Δi*) conditions. (**C**) Histograms showing the statistical analysis of length, G+C content and expression levels of the genes Yra1-enriched genes. Median values are shown (a line represents the genome median). The *p*-value was calculated by Mann-Whitney’s U-test. Asterisks indicate statistically significant differences: *, Ρ<0.05; **, P<0.01; ***, P <0.001 (Mann–Whitney’s U-test) as compared to the genome median. Genes showing expression levels below the median value of the meiotic genes in our experiments in at least 50% of the samples were removed from the comparison analysis.

As Yra1 is an mRNP factor that binds to transcribed genes [10] [35], to gain insight into the effect of *YRA1* overexpression we decided to see if increased amounts of Yra1 protein had any impact on its binding profile along the genome. We performed ChIP-chip experiments using Yra1 proteins expressed from a plasmid at normal levels (*HA-YRA1*) or overexpressed (*HA-YRA1Δi*). Data from asynchronous log-phase cultures were subjected to statistical analysis to obtain the values for Yra1 signal and P-value and to determine the binding profile of Yra1 throughout the genome (see Materials and Methods) (S5 Fig). This revealed that when *YRA1* is overexpressed (*HA-YRA1Δi*) the protein binds to actively transcribed chromatin, it is significantly enriched in ORFs (Fig 6A) and peaks at the 3’ end of genes, as shown in global analysis of the percentage of cluster mapping on different segments along a given ORF (Fig 6B). This profile along the ORF is similar to those of cells expressing Yra1 at normal levels (*HA-YRA1*) and to those reported for Yra1 protein [35].

Moreover, overexpressed Yra1 protein was strongly enriched on the *YRA1* intron (Fig 6A upper panel), consistent with the self-regulated splicing mechanism of *YRA1* gene [35]. More than 50% of the genes (1014 genes) to which Yra1 binds overlap between cells expressing *HA-YRA1* (1749 ORFs) and *HA-YRA1Δi* (1923 ORFs) (Fig 6A; S6A Fig). The analysis of structural and functional features of genes to which Yra1 binds revealed that they were longer and more expressed than the genome average in both normal and overexpressing conditions (Fig 6C). In addition to ORFs, the overexpressed Yra1 protein was also recruited to tRNA and RNAPII-driven non-coding genes, as was previously described for Yra1 protein [35], but it was also enriched at rRNA genes (S6B Fig). Therefore, an excess of Yra1 protein leads to a high accumulation at actively transcribed chromatin, consistent with the role of Yra1 in mRNA biogenesis.

**Fig 7:**
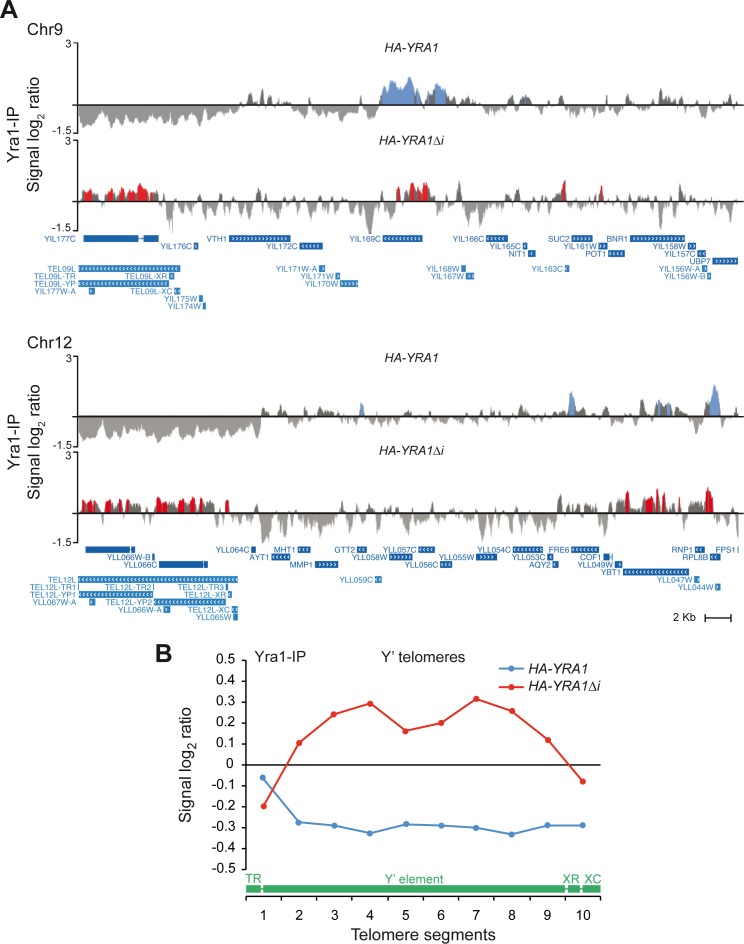
Yra1 recruitment to telomeres under Yra1 WT (*HA-YRA1*) and overexpression (HA-*YRA1Δi*) conditions. (**A**) Fragments of chromosome IX and Chromosome XII are plotted with the signal log_2_ ratio values. (**B**) Composite profile of Yra1 occupancy detected by ChIP-chip across the average Y' element-containing telomeres plotted as signal log_2_ ratio average per each segment. A scheme of a standard Y' telomere is shown. TR, telomeric repeats; XR, X element combinatorial repeat; XC, X element core sequence. Other details as in Fig 6.

Interestingly the genome-wide ChIP-chip data reveals an association of Yra1 to telomeres (Fig 7A). Yra1 binds preferentially to X’ elements in cells without *YRA1* overexpression (HA-*YRA1*), and binds to most of the Y´elements in *YRA1* overexpressing cells (HA-*YRA1Δi*) (S5B Fig). The analysis of Yra1 distribution along the Y’ element-containing telomeres by subdividing them into ten segments of the same length is shown in Fig 7B, where it can be seen the high enrichment of Yra1 along the Y' elements but not at telomeric repeats when it is produced in excess. The genome occupancy of Yra1 protein at telomeric regions and in particular when it is overexpressed, is in agreement with a possible role of this mRNP factor in the maintenance of telomere integrity, at least of Y’-containing telomeres.

We tried to determine the degree of dependency on transcription of Yra1 recruitment to chromatin by treating cells carrying the HA-*YRA1 and* HA-*YRA1Δi* constructs with the RNAP inhibitor actinomycin D (ActD) at concentrations that reduced the RNA levels of RNAPI-, II- and III-transcribed genes to 50–80% of the wild-type levels (S7A Fig) and by comparing recruitment to transcribed versus non-transcribed regions. ChIP followed of qPCR showed that ActD reduced Yra1 occupancy in different transcribed regions tested in the control cells expressing the complete version of *YRA1* (HA-*YRA1*), but had no effect in cells overexpressing *YRA1* (HA-*YRA1Δi*) (S7B Fig). However, recruitment was lower at the non-transcribed meiotic gene *IME1* (not expressed in mitotically dividing cells) with and without ActD. Since ActD inhibits transcription but may not release the transcript and RNAP from the gene, the data are consistent with the conclusion that Yra1 binds preferentially to transcribed chromatin (Fig 6 and S7B Fig).

Since overexpressed Yra1 protein is highly accumulated at active chromatin (Fig 6), we performed a comparative analysis of the transcriptome from cells expressing *GAL*::*YRA1* and *GAL*::*YRA1Δi* constructs in order to know the impact of *YRA1* overexpression on gene expression. Neither specific functional classes of genes nor any DNA damage response genes were deregulated in cells overexpressing *YRA1* (S1 Table), supporting the conclusion that the genome instability associated with overexpression of this mRNP factor is not an indirect consequence. Moreover, microarray analysis of cells overexpressing *YRA1* did not identify a significant enrichment in deregulated genes with specific structural features such as high GC content or length (S1 Table). Consistently, no transcription defect was observed in the reporter *lacZ*-*URA3*, a sequence poorly expressed in several mRNP mutants [48]; (S8 Fig). In summary our analysis revealed that *YRA1* overexpression had no significant impact on global gene expression, although we cannot discard indirect effects derived from the mRNA export defects.

### *YRA1* overexpression leads to DNA replication impairment

**Fig 8:**
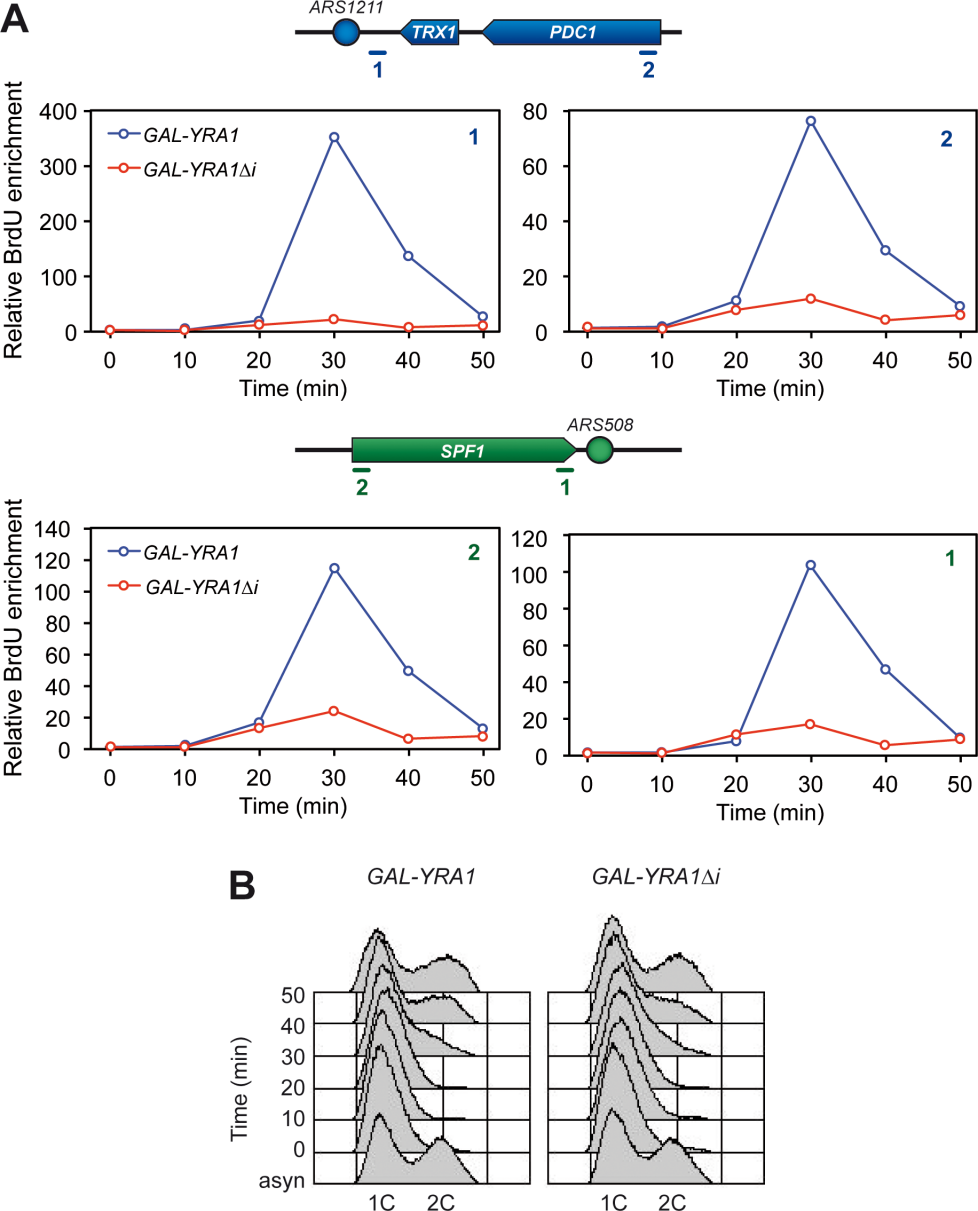
Replication impairment in *YRA1Δi*-overexpressing cells. **(A)** BrdU incorporation in WRBb-9B (WT) cells overexpressing (*GAL-YRA1Δi*) or not (*GAL-YRA1*) *YRA1*. Cells were synchronized in G1 with α-factor in galactose-containing medium for 2 hours and released into fresh galactose-containing medium. BrdU immunoprecipitation was performed at different time points after G1 release, and the resulting DNA was analyzed by qPCR at the early replication origins *ARS1211* (top) and *ARS508* (bottom). Quantification of BrdU incorporation relative to the first time point (without BrdU) is plotted for each region. The plotted values correspond to a representative experiment, while results of two additional experiments are shown in S9 Fig. Schemes of the analyzed regions are shown on top of the graphs. **(B)** FACS analyses from the experiment shown in (A).

As genome instability, measured by DSB accumulation and hyperrecombination, associated with *YRA1* overexpression is transcription dependent (Fig 3), we wondered whether this phenotype was linked to a defect in replication progression, provided that it is known that replication impairment is a major cause of spontaneous DNA breaks. We first observed by FACS that cells overexpressing *YRA1* progressed through S/G2 with a slight delay with respect to wild-type cells (S9 Fig), which suggests that part of its growth defect could be linked to replication impairment. We then analyzed replication by monitoring BrdU incorporation in control (*GAL-YRA1*) and Yra1-overexpressing (*GAL-YRA1Δi*) cells. G1-arrested cells were released from α-factor in galactose-containing medium, to allow entrance into S phase, and subjected to ChIP with anti-BrdU followed of qPCR. BrdU levels peaked at an average of 30 minutes after G1-release in control cells expressing *YRA1* at the early replication origins *ARS1211* and *ARS508*, in which replication and transcription occur head-on (Fig 8 and S10 Fig). In contrast, BrdU incorporation at these sites was dramatically reduced in cells overexpressing Yra1 (*YRA1Δi* construct), indicating that *YRA1* overexpression impairs replication.

**Fig 9:**
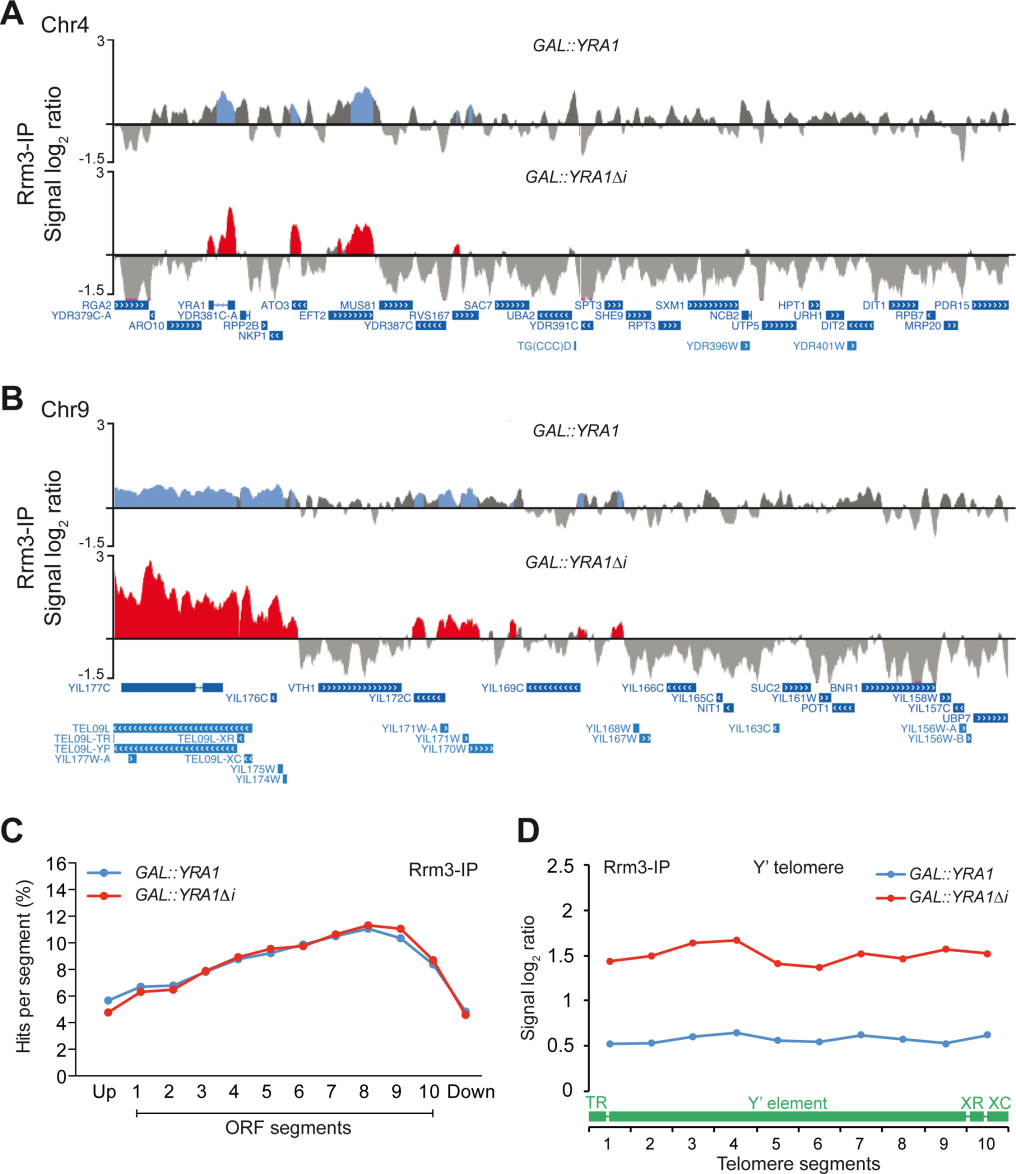
Genome-wide analysis of Rrm3 recruitment under WT (*GAL*::*YRA1*) and overexpression (*GAL*::*YRA1Δi*) conditions. (**A**) Rrm3 recruitment to chromatin in a fragment of chromosome IV is plotted with the signal log_2_ ratio values. (**B**) Rrm3 recruitment to telomeric regions under normal (*GAL*::*YRA1*) and Yra1-overexpressing (*GAL*::*YRA1Δi*) conditions. A fragment of chromosome IX is plotted with the signal log_2_ ratio values. **(C)** Composite profile of Rrm3 occupancy detected by ChIP-chip across the average ORF plotted as percentage of Rrm3 ChIP clusters per segment. (**D**) Composite profile of Rrm3 occupancy detected by ChIP-chip across the average Y' element-containing telomeres plotted as signal log_2_ ratio average per each segment. A scheme of a standard Y' telomere is shown. TR, telomeric repeats; XR, X element combinatorial repeat; XC, X element core sequence. Other details as in Fig 6.

To gain insight into the effect of *YRA1* overexpression on replication fork progression all over the genome we performed ChIP–chip experiments with an Rrm3-Flag fusion protein. Rrm3 is a helicase required for the progression of the RF through obstacles in the DNA, and its accumulation at specific DNA sites has been used to identify RF pauses or stalls [61];[62]. We found that clusters of Rrm3 accumulation were distributed all over the genome both in *GAL*::*YRA1* and *GAL*::*YRA1Δi* expressing cells (Fig 9A, S11 Fig). Detailed analysis of the ORFs in which Rrm3 is accumulated revealed that the helicase peaked at the 3´end of the ORFs (Fig 9C). Cells overexpressing Yra1 accumulated Rrm3 at the same genes as cells expressing normal levels of Yra1 (913 genes), but in addition Rrm3 binds to a new group of 543 genes (up to 1412 total) (S12A Fig), indicating that replication obstacles extend to more genes when Yra1 is overexpressed. 21% of genes occupied by Yra1 showed Rrm3 accumulation (370 of 1749 genes), while this proportion increases to 36% for the *YRA1Δi* constructs (686 of 1923 genes) (S12B Fig). Interestingly, in control cells expressing normal Yra1 levels, only a 6% of the genome occupied by Yra1 is also occupied by Rrm3, whereas this overlap was 40% in Yra1-overexpressing cells (*YRA1Δi* construct). These observations suggest that under Yra1 overexpression Rrm3 accumulates preferentially at Yra1-bound genomic regions, suggesting a link between the stable presence of Yra1 at genome and a negative impact on replication progression. Rrm3 was also detected in more rRNA and tRNA genes (S12C Fig). Moreover, we detected an increase of Rrm3 at Y’ telomeric regions in cells expressing *GAL*::*YRA1Δi*, coincident with the Yra1 presence at these regions (Fig 9B and 9D). Our data suggest that changes in Yra1 stoichiometry could lead to RF progression impairment all over the genome, preferentially at transcribed genes, including Y’ elements.

## Discussion

We overexpressed *YRA1* by transforming yeast cells with *YRA1* intron-less constructs and found that an excess of Yra1 induces DNA damage and hyperrecombination, which in most cases are transcription-dependent and R loop-dependent. In addition, *YRA1* overexpression leads to telomere shortening and a senescence-like phenotype. We provide evidence that *YRA1* overexpression causes a replication impairment determined by the reduction of BrdU incorporation at ARSs in cells overexpressing an intron-less *YRA1Δi* construct. ChIP-chip analysis show that upon overexpression Yra1 is loaded onto transcribed chromatin along the genome and to telomeric regions, where occupancy of the helicase Rrm3 is also increased. The results suggests that genome instability caused by excess of the RNA-binding Yra1 factor is linked to a DNA replication impairment all over the genome including telomeres.

### Replication impairment and transcription-dependent genome instability mediated by RNA:DNA hybrids

It has been reported that overexpressing of ~15% of proteins reduces growth rate [63]. In the case of Yra1 its overexpression could affect numerous processes in the cell, because mRNA export would be affected, which might lead to loss of fitness. Yeast Yra1 protein levels are tightly regulated in a splicing-dependent manner, and its overexpression is toxic as that of other mRNP and export factors partially due to its impact on mRNA export [64] [43]). On the other hand, overexpression of human Yra1 and Hpr1 orthologues ALY and THOC1, respectively, in a broad range of tumors, highlights the ability of these proteins to undergo stoichiometry changes in tumor cells [44,45]. Moreover, ALY depletion in cells by siRNA leads to DNA damage as determined by an increase in γH2AX foci formation [19]. Interestingly, our results indicate that *YRA1* overexpression also leads to genome instability (Figs 1–4), a hallmark of cancer cells. This instability, detected by hyper-recombination, is dependent on transcription and R loop accumulation. The genetic and physical interaction of Yra1 with Sub2 and THO RNA biogenesis and export factors, which suppress RNA-mediated genome instability by partially preventing R-loop formation, is consistent with the observation that Yra1 overexpression leads to a hyper-recombination phenotype of a similar molecular nature to those of *tho* and *sub2* mutants. Nevertheless, co-overexpression of Yra1 and Sub2 proteins (S1 Fig) indicated that genome instability associated with Yra1 excess is not due to a potential sequestering of THO-Sub2 complex subunits by an excess of free Yra1. Thus, the effect of *YRA1* overexpression on genome instability seems to be independent of Sub2, which is consistent with the observation that *YRA1* overexpression leads to telomere shortening whereas *SUB2* overexpression has been shown not to affect telomere length [65].

**Fig 10:**
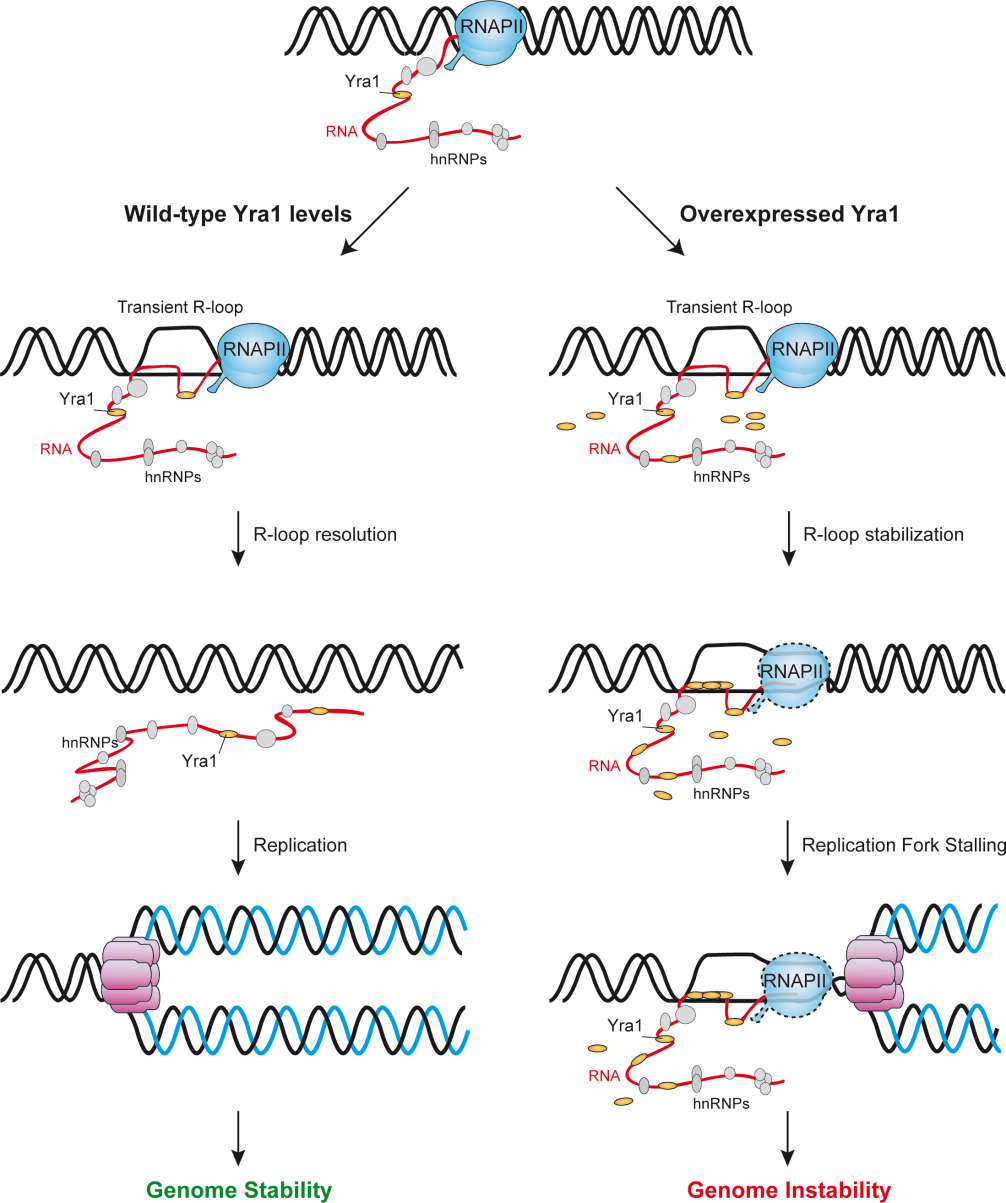
A model to explain how an excess of Yra1 could lead to replication impairment and genome instability. In yeast cells, transient and unstable RNA-DNA hybrids could form co-transcriptionally at specific regions, but they would be efficiently resolved if Yra1 is present at wild-type levels. However, under Yra1 overexpression, the excess of Yra1 could bind either to the RNA moiety of RNA:DNA hybrids or to the nascent RNA as it comes out of the RNAPII, stabilizing a co-transcriptional structure, mainly an RNA:DNA hybrid, able to block the progression of the replication fork. A subsequent collapse of the fork would lead to single- or double-strand breaks and genome instability in the form of DNA recombination. Telomere shortening could be a manifestation of this phenomenon or could be due to the possibility that an excess of Yra1 could bind to the TLC1 RNA interfering with the telomerase activity.

The hyper-recombination conferred by Yra1 overexpression may require the RNA- and CTD-binding domains of Yra1 (Fig 3C), which would suggest that the ability of Yra1 to bind RNA and active chromatin might be critical for the phenotype caused by its overexpression. Yra1 was initially identified on the basis of its potent RNA annealing activity *in vitro* [66], and binds to RNAPII-transcribed genes with a localization bias toward the 3´ends of genes as is the case of the profile of the RNAPII CTD Ser2-P mark and a number of transcription elongation and mRNP factors, including the THO complex with which Yra1 interacts [67] [68] [35] [25] [10]. Yra1 ChIP experiments performed in ActD-treated cells (S7B Fig) are in agreement with the idea that Yra1 is recruited to transcribed chromatin. An excess of Yra1 could in principle interfere with the transcription process, but this does not seem to be the case since the binding profile of Yra1 along the ORFs was quite similar regardless of whether or not *YRA1* was overexpressed (Fig 6) and a significant impact on gene expression was not revealed by microarray analysis (S1 Table). It is likely that the excess of Yra1 bound to the transcribed chromatin could affect the replication process. In this sense, Yra1 has been previously identified as a partner of Dia2, a protein associated with replication origins [69], although the functional relationship between these two observations is unclear yet. In any case, our results of BrdU incorporation support that *YRA1* overexpression leads to replication progression impairment (Fig 8). Moreover, under *YRA1* overexpression, the genome-wide profile of Rrm3 helicase occupancy, used as a marker of replication forks, supports this hypothesis (Fig 9). In Yra1-overexpressing cells there is an enrichment of Rrm3 in a significantly higher number of ORFs, including those of the highly transcribed ribosomal protein genes, in which Yra1 is also enriched (Fig 9, S6 and S12 Fig). Genome-wide analyses reveal that Rrm3 accumulates preferentially at Yra1-bound genomic regions when it is overexpressed (S12B Fig). Thus, our data suggest that the cellular stoichiometry of the hnRNP Yra1 is relevant for genome integrity and replication in a transcription-dependent and R loop-dependent manner. It is possible that the excess of Yra1 causes tightly bound transcription machinery at transcribed genes that may transiently cause a block of the replication fork as a source of genome instability [17]. Recent evidence indicates that R-loops might also be formed in human cells, for which DNA repair proteins such as BRCA1, BRCA2 and other Fanconi Anemia factors could play a kind of back-up system to remove then [70,71,72,73]. An excess of Yra1, being Yra1 an RNA-binding protein, could also bind to co-transcriptional RNA:DNA hybrids transiently formed, therefore promoting a tighter and more stable structure able to block the passage of the replication fork and responsible for the instability (Fig 10).

### Telomere shortening

Our results suggest that Yra1 could have a role in telomere maintenance, as deduced from the fact that it binds to telomeres and its overexpression causes telomere shortening (Figs 5 and 7). Several factors involved in different steps of transcription and chromatin remodeling, such as components of the RSC, Mediator, Paf1 and THO complexes, Set1, RNAPII and factors involved in phosphorylation of its CTD, and some splicing and 3’-end processing factors have been identified in different screenings for telomere-length alterations in yeast [74] [75] [76]. Moreover, other RNA binding proteins also bind to telomeres [77];[65]. In addition, DDX39, a human DEAD-box RNA helicase with a high homology to UAP56/Sub2, binds to the shelterin subunit TRF2 implicated in telomere protection, and its depletion activates the DNA-damage response and leads to aberrant telomere structures in human cells [78]. However, nuclear factors may also affect telomere integrity by altering structural telomeric intermediates required for telomere function. In this context, deregulated TERRA transcription has been associated with telomere shortening, senescence and disease (reviewed in [79]). There is evidence that RNA:DNA hybrids formed at telomeres can regulate telomere dynamics and accelerate senescence, and that mutations in THO subunits enhance these features [80]; [81] [82]. Here we show that the stoichiometry of specific RNA-binding factors such as Yra1 at telomeres is critical for telomere homeostasis. However, at this point we do not know whether this is due to a major presence at the telomeric repeats; therefore, it is unlikely that telomere shortening is due to direct action of Yra1 on TERRA RNA or RNA:DNA hybrids. We need to know if all telomeres, whether or not containing subtelomeric Y’ regions, shorten, and what causes this shortening. It is likely that, once cells reach a minimum telomere size for survival, this is maintained by a stable equilibrium between the action of telomerase and recombination. We cannot discard that the excess of Yra1 could bind to the *TLC* RNA interfering with the telomerase activity, a possibility that needs to be addressed experimentally. However, according to the consequences of Yra1 excess in the rest of the genome, it is likely that replication impairment caused by the transcription apparatus and/or RNA:DNA hybrids (Fig 10) also contributes to telomere shortening.

Finally, the senescence-like phenotype could be the result of DNA instability generated by replication impairment after several generations. Interestingly, a reduced life-span phenotype associated with increased genome instability has been reported in *hpr1Δ*, a mutant of the THO complex [83]. It is possible that the growth defect and telomere shortening linked to the overexpression of Yra1 are related to replication impairment, consistent with the increase of Rrm3 occupancy at Y’ telomeric regions (Fig 9). However, the cause-effect relationship between telomere shortening and the senescence-like phenotype is yet to be resolved.

## Materials and Methods

### Yeast strains and plasmids

Yeast strains used are listed in S2 Table. *YRA1* gene (*YRA1*) and *YRA1* cDNA (*YRA1Δi*) were cloned in centromeric plasmids pCM184, pCM189 [84] and pRS413GAL [85] in order to achieve different *YRA1* expression levels, to obtain plasmids pCM184*tet*::YRA1, pCM184*tet*::YRA1Δi, pCM189*tet*::YRA1, pCM189*tet*::YRA1Δi, pRS413GAL::YRA1 and pRS413GAL::YRA1Δi. Plasmids pHA-YRA1, pHA-YRA1Δi, pHA-YRA1ΔN and pHA-YRA1ΔRBD carried different versions of YRA1 tagged in its N-terminal end with HA epitope [27]. Plasmids pRS314L, pRS316L, pRS314LY, pRS316LY and pRS316-LYΔNS [50], pRS314L-*lacZ*, pRS314GL-*lacZ* [51,85] pGL-*rib*^*m*^, pGL-*Rib*+, pGAL:RNH1 [18] and p413GALAID [53] were used to determine recombination frequencies. Plasmid pWJ1344 containing the tagged *RAD52*-YFP fusion [86] was used for DNA-damage analysis. Plasmid pCM184-LAUR [48] was used for the analysis of mRNA expression levels.

### Analyses of Rad52-YFP foci and recombination frequencies

Spontaneous Rad52-YFP foci from mid-log growing cells carrying plasmid pWJ1344 were visualized and counted by fluorescence microscopy [86]. Experiments were performed in cells after 15 hours in medium containing galactose to achieve the overexpression of both Yra1 and Rnh1. Cells transformed with Recombination frequencies were determined as described [87]. For each strain, the recombination frequencies are given as the average and standard deviation of the median recombination frequency value obtained from fluctuation tests performed in 3–4 different transformants using 6 independent colonies per transformant. Recombinants were selected as Leu+ colonies for the plasmid containing *LEU2* truncated repeat systems. Recombination analyses for the chromosomal *leu2-k*::*ADE2-URA3*::*leu2-k* system (Lk-AU) [88] were performed in wild-type and congenic mutants using 6 to 12 independent colonies grown in synthetic complete medium SC, and recombinants were selected in SC + FOA.

### Yeast passaging and telomere length analysis

Transformants were streaked on SC medium with 2% galactose supplemented with 0.5% glucose for 3 days before being passaged onto plates with fresh media. After each passage cells were growth to obtain DNA genomic for Southern analysis.

Analysis of young and aged cultures were performed with cells grown in liquid SC medium with 2% galactose supplemented with 0.5% glucose. In order to achieve a high amount of cell divisions, cultures were diluted after ten generations in fresh media, after which they were diluted in several rounds successively and maintained in exponential phase during 80 generations. Generations were estimated indirectly by measuring optical density of the cultures. Samples (2–5 ml) were collected from cultures before diluting in fresh media at every step. After 70–80 generations, samples were taken for recombination test, DNA and RNA extraction.

After extraction, DNA (2–3 μg) was digested with *Xho*I overnight, separated by electrophoresis (16–18 h at 4 V/cm using 1% 20–25 cm agarose gel in TBE, and transferred to Hybond-N membranes [75]. Terminal restriction fragments were visualized by hybridization with ^32^P-labeled Y′-probes.

### Microarray gene expression analysis

Microarray analysis of total RNA was performed using GeneChIP Yeast Genome 2.0 Array Affymetrix as previously described [68]. Briefly, total RNA was isolated from wildtype cells transformed with the *GAL*:*YRA1* or *GAL1*::*YRA1Δi* constructs growing in raffinose and shifted for four hours to galactose (2%). RNA was extracted using the RNeasy Midi kit (Qiagen). RNA levels for all yeast genes were determined using Affymetrix microarray scanner. Microarray was conducted in triplicate and the values presented represent the average of these three determinations. The expression data can be accessed at Gene Expression Omnibus (GSE68488; GSE68487).

### Analysis of transcription by RT-qPCR

Total RNA was extracted from exponentially growing cells in SC-Trp medium and treated or not with 10 μg/ml of ActD for 2 hours. RNA was extracted with acid phenol, treated with DNase I (Invitrogen), and cDNA was obtained by the Superscript® III First-Strand Synthesis System (Invitrogen) from 1 μl of RNA following the manufacturer’s instructions. qPCR was performed and relative RNA levels were determined by absolute quantitation normalized to the total amount of DNA extracted from the same cultures. Primers used are listed in S3 Table. Average and SD of three independent experiments are shown.

### ChIP experiments

Recruitment of HA-*YRA1* and HA-*YRA1Δi* to chromatin was analyzed in exponentially growing cells in SC-Trp medium and treated or not with 10 μg/ml of ActD for 2 hours. ChIP analyses were performed as previously described [89] with some modifications: 1.5 mg/mL pronase was used instead of proteinase K to remove proteins in the de-crosslinking step; and the QIAquick PCR purification kit (Qiagen) was used for the last DNA purification step. Anti-HA tag antibody (ChIP Grade, Abcam) was used and qPCR was performed as described [90]. The represented values were calculated as the log_2_ ratio between treated and untreated cultures. Primers used for qPCR are listed in S3 Table. Average and SD of three independent experiments are shown.

### BrdU incorporation assays

Analysis of replication by BrdU incorporation was performed as previously described [91]. Briefly, strains carrying the mutation *bar1Δ*, several copies of the Herpes simplex thymidine kinase (TK) under the control of the strong constitutive *GPD* promoter, and the constructions *GAL-YRA1* or *GAL-YRA1Δi* were grown in 2% raffinose-containing medium lacking His, added 2% galactose and synchronized in G1 with α-factor for 2 hours, and released into fresh 2% raffinose-2% galactose-containing medium. 200 μg/ml BrdU was then added and culture samples were taken at the indicated time points. BrdU-IP was carried out as described [89], with some modifications. Sodium Azide (0.1%) was added to each sample and cells were broken at 4°C in lysis buffer (50 mM HEPES-KOH pH 7.5, 140 mM NaCl, 1 mM EDTA, 1% triton X-100, 0.1% sodium deoxycholate) and sonicated. Immunoprecipitation was performed using anti-BrdU antibody (MBL) attached to magnetic beads coated with Protein A (Invitrogen). Input and precipitated DNA were analyzed by qPCR and relative BrdU incorporation at a given region was calculated by absolute quantification relative to the signal of the first time point (without BrdU). Primers used for qPCR are listed in S3 Table.

### ChIP-chip experiments

*S*. *cerevisiae* oligonucleotide tiling microarrays were provided by Affymetrix (GeneChip *S*. *cerevisiae* Tiling 1.0R array). The high-density oligonucleotide arrays used allows the analysis of yeast chromosomes at a 300-bp resolution, each of the 300-bp region being covered by at least 60 probes. ChIP-chip of asynchronously growing cells was carried out as described [92] [93]. For immunoprecipitation with Rrm3-FLAG, cells growing in raffinose were shifted for four hours to galactose (2%) to overexpress *YRA1*. Overexpression after 4 hours was checked by Northern. Briefly, 1.5x10^7^ cells were disrupted by multi-beads shocker (MB400U, Yasui Kikai, Japan), which maintained cells precisely at lower than 4°C during disruption. Anti-HA tag antibody (ChIP Grade, Abcam) and anti-FLAG antibody M2 (Sigma-Aldrich) were used for ChIP. ChIP DNA was purified and amplified by random priming using a WGA2 kit (Sigma- Aldrich) and following the manufacturer’s procedure. A total of 4 μg of amplified DNA was digested with DNaseI to a mean size of 100 bp and the purified DNA fragments were end-labelled with biotin-N6-ddATP23. The ChIP-chip data can be accessed at Gene Expression Omnibus (GSE68488; GSE68486).

### Statistical analysis of genome-wide data

Microarray expression data were normalized by RMA (robust microarray average) and statistically analyzed by LIMMA (linear models for microarray analysis), comparing the mutant expression profile with its isogenic wild-type strain. The genes showing at least a 1.5-fold expression change with a P-value < 0.01 with a false discovery rate (FDR) corrections were considered as significantly altered.

ChIP–chip data were analyzed using the Tiling Array suite (TAS) software from Affymetrix. For each probe position, TAS produces the signal and the change P-value, taking into account the probes localized within a given band-width around the inspected probe. Protein chromosomal distribution was then analyzed by detecting binding clusters, which were defined as ranges within the chromosome respecting the following conditions: estimated signal (IP/SUP-binding ratio) positive in the whole range, P-value < 0.01, minimum run of 100 bp, and maximum gap of 250 bp.

The results were visualized with the UCSC Genome Browser, developed and maintained by the Genome Bioinformatics Group (Center for Biomolecular Science and Engineering at the University of California at Santa Cruz; http://genome.ucsc.edu/). Distribution of binding sites along genes was carried out as previously described [68].

### Miscellanea

Analysis of sensitivity to genotoxic agents, Southern, Northern, Western and FACS using a FACScalibur Becton Dickinson machine were performed using standard procedures. Primers used are detailed in S3 Table. Antibodies anti–HA (Abcam) and anti-actin (Sigma) were used in western analysis.

## Supporting Information

S1 Fig (**A**) Effect of *YRA1* overexpression together with or without *SUB2* overexpression. Ten-fold serial dilutions of WT cells transformed with either *GAL*::*YRA1Δi*, *GAL*::*SUB2 or GAL*::*YRA1Δi and GAL*::*SUB2* constructs and plated on minimal selective medium with 2% galactose (Gal) and different amounts of glucose (Glu). Photographs were taken after 3 days of growth at 30°C. (**B**) Recombination analysis of the plasmid-borne recombination systems LY in WT cells transformed with either *tet*:*YRA1*, *tet*:*YRA1Δi*, *tet*::*SUB2* or *tet*::*SUB2* and *tet*::*YRA1Δi* constructs. Gray boxes represent *LEU2* repeats. Arrows indicate the transcripts produced.
(PDF)

S2 Fig Relative expression of HA-tagged Yra1 proteins. Western blot analysis of cells transformed with HA-YRA1, HA-YRA1ΔI and HA-YRA1ΔRBDΔi constructs is plotted.
(PDF)

S3 Fig Effect of AID overexpression on the recombination frequency of the LYΔNS system in cells transformed with *tet*:*YRA1* or *tet*:*YRA1Δi* constructs. The *hpr1Δ* mutant was included as positive control. Experiments were performed in 2% galactose to allow expression of the direct repeats.
(PDF)

S4 Fig Southern analysis of genomic DNA from different *yra1* mutants. Strains carrying *YRA1* deletion complemented with the *YRA1* gene (*YRA1*), the mutant allele *yra1-1*, the complete cDNA (*YRA1Δi*) or the cDNA lacking the RBD domain (*YRA1ΔRBDΔi*) were grown in SC-Ura or SC-Trp medium. Three different transformants of each strain were analyzed by Southern blot. Sub-telomeric and telomeric fragments are visualized using a telomeric-specific probe (Y’ probe). Other details as in Fig 5.
(PDF)

S5 Fig Genomic view of Yra1 recruitment under Yra1 wild-type (*HA-YRA1*) and Overexpression (*HA-YRA1Δi*) conditions. A representation of each chromosome with the signal log_2_ ratio values for the significant ChIP-chip clusters is plotted. The X-axis shows chromosomal coordinates in kb. Positions of centromeres are indicated as open circles.
(PDF)

S6 Fig (**A**) Venn diagrams showing the overlap between genes sets with significant Yra1 binding in the different ChIP-chip experiments. (**B**) Yra1 cluster distribution at ARSs, centromeres, introns, ncRNA, transposable elements, RNAPIII genes, snoRNA/snRNA and telomeres under wild-type (HA-*YRA1*) and overexpression (HA-*YRA1*Δi) conditions.
(PDF)

S7 Fig Yra1 binding to chromatin upon transcription inhibition. **(A)** RT-qPCR analysis of transcription levels of W303-1A (WT) cells expressing HA-*YRA1* and HA-*YRA1Δi* constructs, treated or not with actinomycin D (ActD) for 2 hours. **(B)** ChIP and qPCR analysis in specific genomic regions of Yra1 in the same strains and conditions as in (A). Average and SD of three independent experiments are shown. Asterisks indicate statistically significant differences between the indicated conditions, according to Student's t-tests (*, Ρ< 0.05).
(PDF)

S8 Fig Northern of *lacz-URA3* system in cells expressing *GAL*::*YRA1* or *GAL*::*YRA1Δi*. (PDF)

S9 Fig FACS profiles from WT cells transformed with either the *GAL*::*YRA1Δi* construct or the empty vector. Cells were synchronized in G1 with α-factor and released at 30°C.
(PDF)

S10 Fig BrdU incorporation analysis for two independent experiments in WRBb-9B (WT) cells overexpressing (*GAL-YRAΔi*) or not (*GAL-YRA1*) *YRA1*. Other details as in Fig 8.
(PDF)

S11 Fig Genomic view of Rrm3 recruitment under wild-type (*GAL*::*YRA1*) and Overexpression (*GAL*::*YRA1Δi*) conditions. A representation of each chromosome with the signal log_2_ ratio values for the significant Rrm3 binding clusters is plotted. The X-axis shows chromosomal coordinates in kb. Centromeres are indicated as open circles.
(PDF)

S12 Fig (**A**) Venn diagrams showing the overlap between genes with significant Rrm3 binding in the different ChIP-chip experiments. (**B**) Venn diagrams showing the overlap between genes with significant Yra1 and Rrm3 binding in different ChIP-chip experiments. **(C)** Rrm3 cluster distribution at ARSs, centromeres, introns, ncRNA, transponible elements, RNAPIII genes, snoRNA/snRNA and telomeres under wild-type (GAL::*YRA1*) and Overexpression (GAL::*YRA1Δi*) conditions.
(PDF)

S1 Table Microarray gene expression analysis in cells overexpressing *YRA1*. Top genes whose expression levels are up-regulated and down-regulated in YRA1-overexpressing cells (*GAL*::*YRA1Δi*).
(PDF)

S2 Table Strains used in this study. (PDF)

S3 Table Primers used in this study. (PDF)
