# Supplementary material for: Excess of Yra1 RNA-Binding Factor Causes Transcription-Dependent Genome Instability, Replication Impairment and Telomere Shortening
Source: PLoS Genet. 2016 Apr 1;12(4):e1005966. doi: 10.1371/journal.pgen.1005966 (PMC4818039; doi:10.1371/journal.pgen.1005966)
Supplement: S1 Fig — (A) Effect of YRA1 overexpression together with or without SUB2 overexpression. Ten-fold serial dilutions of WT cells transformed with either GAL::YRA1Δi, GAL::SUB2 or GAL::YRA1Δi and GAL::SUB2 constructs and plated on minimal selective medium with 2% galactose (Gal) and different amounts of glucose (Glu). Photographs were taken after 3 days of growth at 30°C. (B) Recombination analysis of the plasmid-borne recombination systems LY in WT cells transformed with either tet:YRA1, tet:YRA1Δi, tet::SUB2 or tet::SUB2 and tet::YRA1Δi constructs. Gray boxes represent LEU2 repeats. Arrows indicate the transcripts produced. (PDF) [file pgen.1005966.s001.pdf]

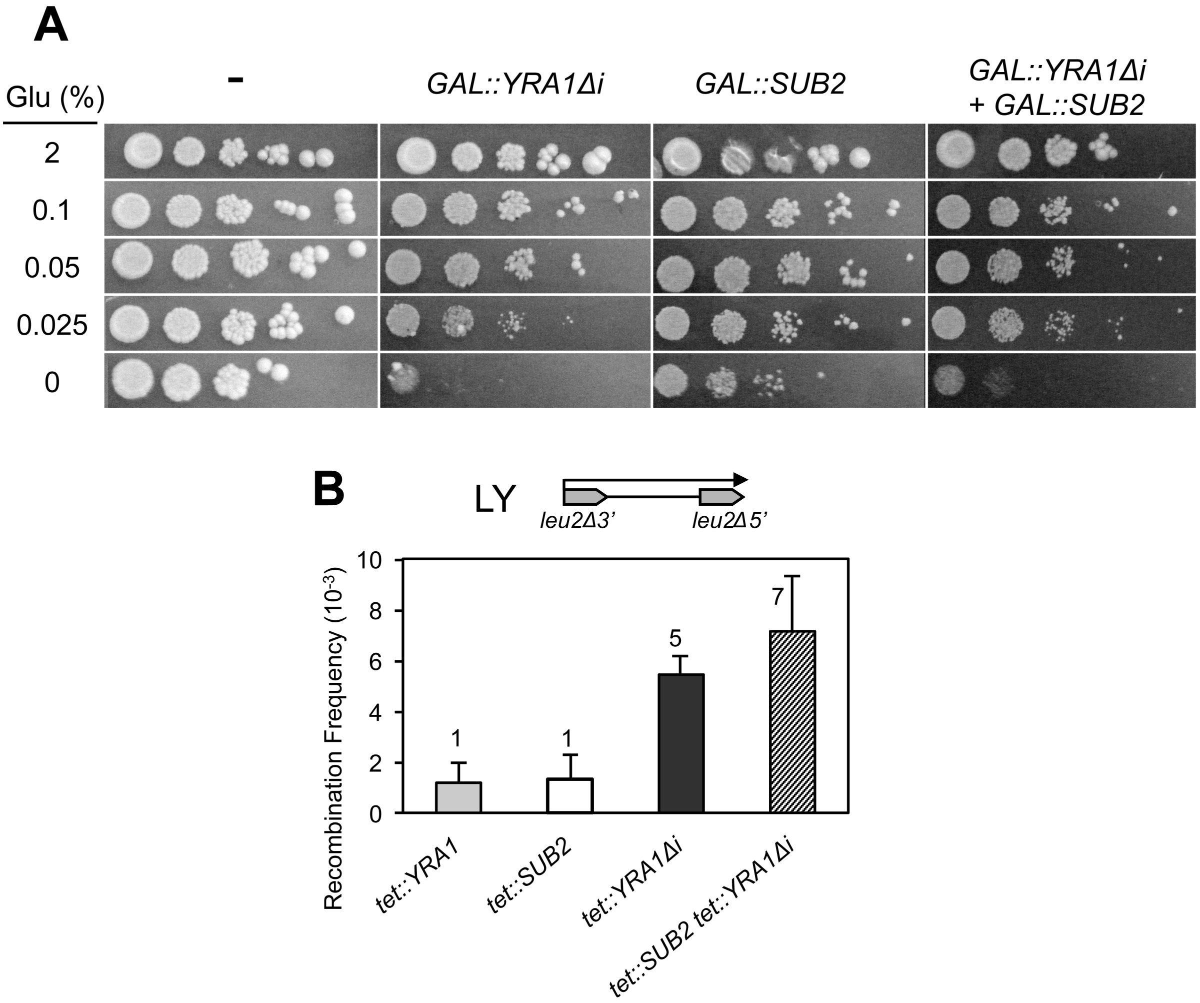

S1 Figure (A) Effect of *YRA1* overexpression together with or without *SUB2* overexpression. Ten-fold serial dilutions of WT cells transformed with either *GAL::YRA1Δi*, *GAL::SUB2* or *GAL::YRA1Δi* and *GAL1::SUB2* constructs and plated on minimal selective medium with 2% galactose (Gal) and different amounts of glucose (Glu). Photographs were taken after 3 days of growth at 30ºC. **(B)** Recombination analysis of the plasmid-borne recombination systems LY in WT cells transformed with either *tet:YRA1*, *tet:YRA1Δi*, *tet::SUB2* or *tet::SUB2* and *tet::YRA1Δi* constructs. Gray boxes represent *LEU2* repeats. Arrows indicate the transcripts produced.
