# Supplementary material for: Excess of Yra1 RNA-Binding Factor Causes Transcription-Dependent Genome Instability, Replication Impairment and Telomere Shortening
Source: PLoS Genet. 2016 Apr 1;12(4):e1005966. doi: 10.1371/journal.pgen.1005966 (PMC4818039; doi:10.1371/journal.pgen.1005966)
Supplement: S2 Fig — Western blot analysis of cells transformed with HA-YRA1, HA-YRA1ΔI and HA-YRA1ΔRBDΔi constructs is plotted. (PDF) [file pgen.1005966.s002.pdf]

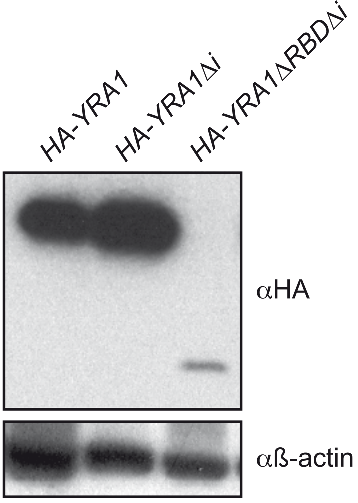

S2 Figure. Relative expression of HA-tagged Yra1 proteins. Western blot analysis of cells transformed with HA-*YRA1*, HA-*YRA1Δi* and HA-*YRA1ΔRBDΔi* constructs is plotted.
