# Supplementary material for: Excess of Yra1 RNA-Binding Factor Causes Transcription-Dependent Genome Instability, Replication Impairment and Telomere Shortening
Source: PLoS Genet. 2016 Apr 1;12(4):e1005966. doi: 10.1371/journal.pgen.1005966 (PMC4818039; doi:10.1371/journal.pgen.1005966)
Supplement: S3 Fig — The hpr1Δ mutant was included as positive control. Experiments were performed in 2% galactose to allow expression of the direct repeats. (PDF) [file pgen.1005966.s003.pdf]

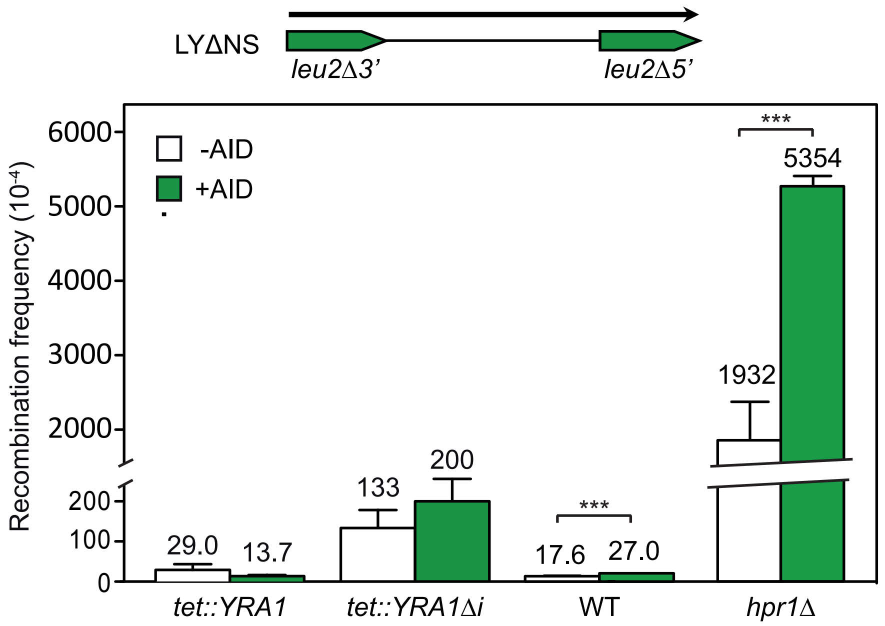

S3 Figure. Effect of AID overexpression on the recombination frequency of the LYΔNS system in cells transformed with *tet:YRA1* or *tet:YRA1Δi* constructs. The *hpr1Δ* mutant was included as positive control. Experiments were performed in 2% galactose to allow expression of the direct repeats.
