# Supplementary material for: Excess of Yra1 RNA-Binding Factor Causes Transcription-Dependent Genome Instability, Replication Impairment and Telomere Shortening
Source: PLoS Genet. 2016 Apr 1;12(4):e1005966. doi: 10.1371/journal.pgen.1005966 (PMC4818039; doi:10.1371/journal.pgen.1005966)
Supplement: S4 Fig — Strains carrying YRA1 deletion complemented with the YRA1 gene (YRA1), the mutant allele yra1-1, the complete cDNA (YRA1Δi) or the cDNA lacking the RBD domain (YRA1ΔRBDΔi) were grown in SC-Ura or SC-Trp medium. Three different transformants of each strain were analyzed by Southern blot. Sub-telomeric and telomeric fragments are visualized using a telomeric-specific probe (Y’ probe). Other details as in Fig 5. (PDF) [file pgen.1005966.s004.pdf]

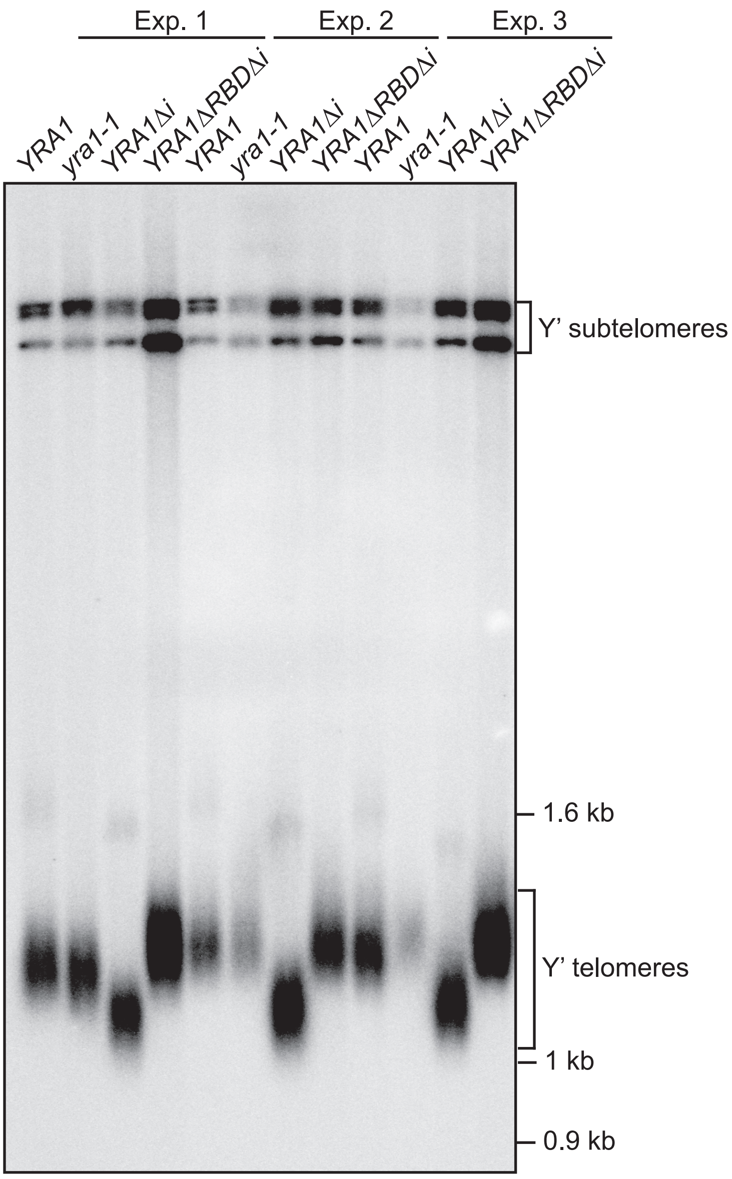

S4 Figure. Southern analysis of genomic DNA from different *yra1* mutants. Strains carrying *YRA1* deletion complemented with the *YRA1* gene (*YRA1*), the mutant allele *yra1-1*, the complete cDNA (*YRA1Δi*) or the cDNA lacking the RBD domain (*YRA1ΔRBDΔi*) were grown in SC-Ura or SC-Trp medium. Three different transformants of each strain were analyzed by Southern blot. Sub-telomeric and telomeric fragments are visualized using a telomeric-specific probe (Y' probe). Other details as in Fig 5.
