# Supplementary material for: Excess of Yra1 RNA-Binding Factor Causes Transcription-Dependent Genome Instability, Replication Impairment and Telomere Shortening
Source: PLoS Genet. 2016 Apr 1;12(4):e1005966. doi: 10.1371/journal.pgen.1005966 (PMC4818039; doi:10.1371/journal.pgen.1005966)
Supplement: S5 Fig — A representation of each chromosome with the signal log2 ratio values for the significant ChIP-chip clusters is plotted. The X-axis shows chromosomal coordinates in kb. Positions of centromeres are indicated as open circles. (PDF) [file pgen.1005966.s005.pdf]

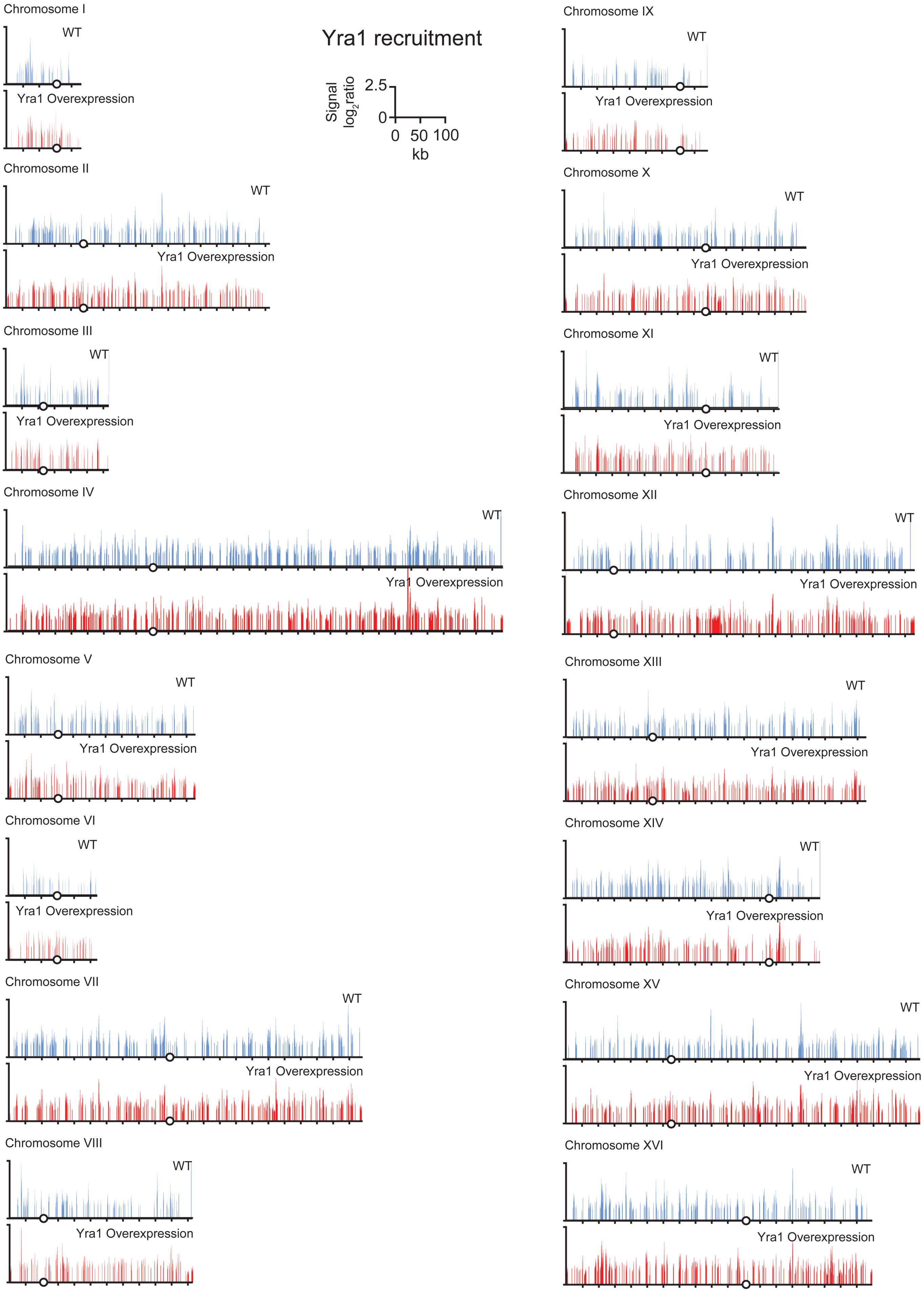


**S5 Figure.** Genomic view of Yra1 recruitment under Yra1 wild-type (HA-*YRA1*) and Overexpression (HA-*YRA1∆i*) conditions. A representation of each chromosome with the signal $\log_2$ ratio values for the significant ChIP-chip clusters is plotted. The X-axis shows chromosomal coordinates in kb. Positions of centromeres are indicated as open circles.
