# Supplementary material for: Excess of Yra1 RNA-Binding Factor Causes Transcription-Dependent Genome Instability, Replication Impairment and Telomere Shortening
Source: PLoS Genet. 2016 Apr 1;12(4):e1005966. doi: 10.1371/journal.pgen.1005966 (PMC4818039; doi:10.1371/journal.pgen.1005966)
Supplement: S6 Fig — (A) Venn diagrams showing the overlap between genes sets with significant Yra1 binding in the different ChIP-chip experiments. (B) Yra1 cluster distribution at ARSs, centromeres, introns, ncRNA, transposable elements, RNAPIII genes, snoRNA/snRNA and telomeres under wild-type (HA-YRA1) and overexpression (HA-YRA1Δi) conditions. (PDF) [file pgen.1005966.s006.pdf]

A

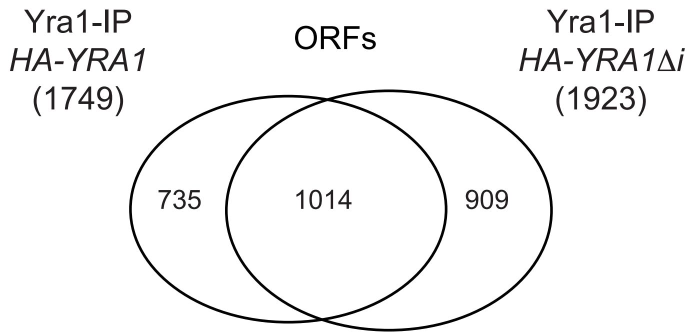

B

Yra1-IP Recruitment

	HA-YRA1	*HA-YRA1Δi*	Total
ARS	18	20	336
Centromeres	1	2	16
Introns	22	16	376
ncRNA	2	1	15
Retrotransposon	0	39	50
Transposable element	0	71	89
rRNA	0	21	27
snoRNA/snRNA	22	10	83
tRNA	7	3	299
Telomeres	20	20	63
X'Element	13	2	60
Y'Element	2	18	19

S6 Figure. (A) Venn diagrams showing the overlap between genes sets with significant Yra1 binding in the different ChIP-chip experiments. **(B)** Yra1 cluster distribution at ARSs, centromeres, introns, ncRNA, transposable elements, RNAPIII genes, snoRNA/snRNA and telomeres under wild-type (HA-*YRA1*) and overexpression (HA-*YRA1Δi*) conditions.
