# Supplementary material for: Excess of Yra1 RNA-Binding Factor Causes Transcription-Dependent Genome Instability, Replication Impairment and Telomere Shortening
Source: PLoS Genet. 2016 Apr 1;12(4):e1005966. doi: 10.1371/journal.pgen.1005966 (PMC4818039; doi:10.1371/journal.pgen.1005966)
Supplement: S7 Fig — (A) RT-qPCR analysis of transcription levels of W303-1A (WT) cells expressing HA-YRA1 and HA-YRA1Δi constructs, treated or not with actinomycin D (ActD) for 2 hours. (B) ChIP and qPCR analysis in specific genomic regions of Yra1 in the same strains and conditions as in (A). Average and SD of three independent experiments are shown. Asterisks indicate statistically significant differences between the indicated conditions, according to Student's t-tests (*, Ρ< 0.05). (PDF) [file pgen.1005966.s007.pdf]

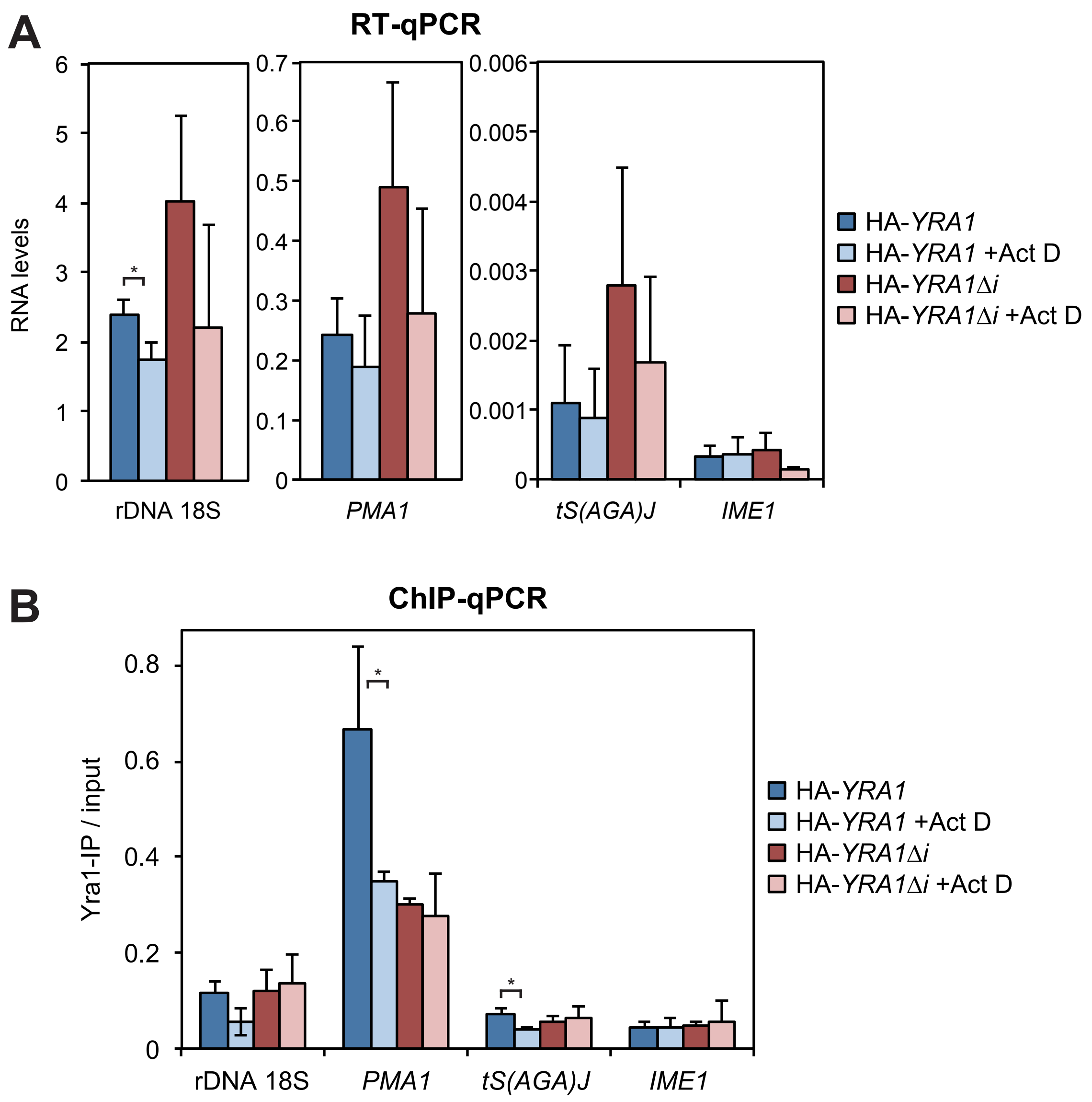

S7 Figure. Yra1 binding to chromatin upon transcription inhibition. **(A)** RT-qPCR analysis of transcription levels of W303-1A (WT) cells expressing HA-*YRA1* and HA-*YRA1Δi* constructs, treated or not with actinomycin D (ActD) for 2 hours. (B) ChIP and qPCR analysis in specific genomic regions of Yra1 in the same strains and conditions as in (A). Average and SD of three independent experiments are shown. Asterisks indicate statistically significant differences between the indicated conditions, according to Student's *t*-tests (*, P< 0.05).
