# Supplementary figures and images for: Excess of Yra1 RNA-Binding Factor Causes Transcription-Dependent Genome Instability, Replication Impairment and Telomere Shortening
Source: PLoS Genet. 2016 Apr 1;12(4):e1005966. doi: 10.1371/journal.pgen.1005966 (PMC4818039; doi:10.1371/journal.pgen.1005966)

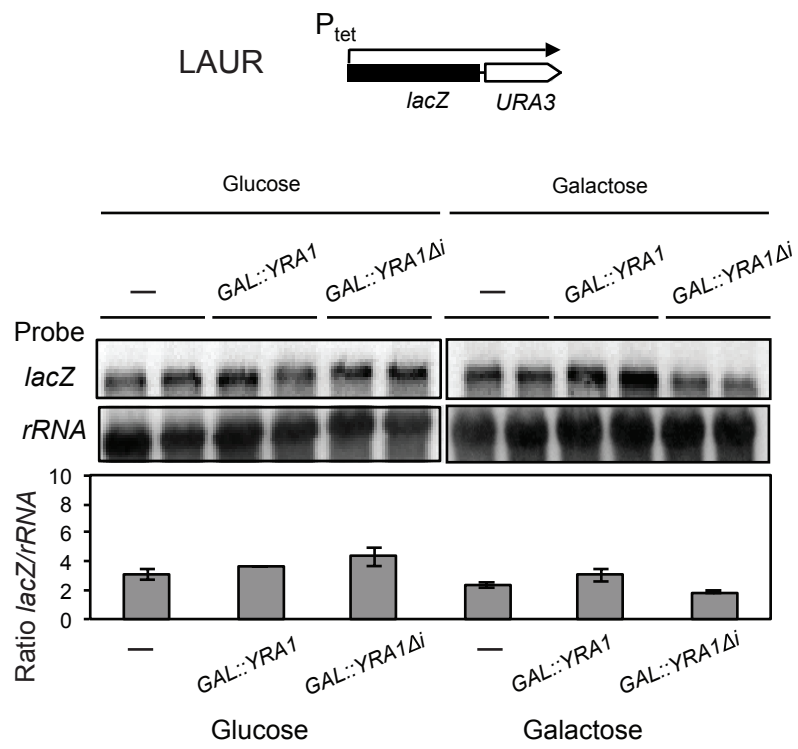

**S8 Figure.** Northern of *lacZ-URA3* system in cells expressing *GAL::YRA1* or *GAL::YRA1Δi*.

Supplement: S8 Fig — (PDF) [file pgen.1005966.s008.pdf]
