# Supplementary material for: Excess of Yra1 RNA-Binding Factor Causes Transcription-Dependent Genome Instability, Replication Impairment and Telomere Shortening
Source: PLoS Genet. 2016 Apr 1;12(4):e1005966. doi: 10.1371/journal.pgen.1005966 (PMC4818039; doi:10.1371/journal.pgen.1005966)
Supplement: S9 Fig — Cells were synchronized in G1 with α-factor and released at 30°C. (PDF) [file pgen.1005966.s009.pdf]

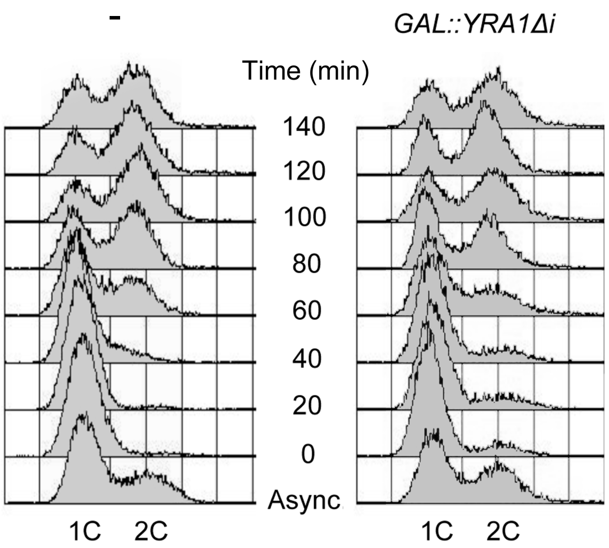

S9 Figure. FACS profiles from WT cells transformed with either the *GAL::YRA1Δi* construct or the empty vector. Cells were synchronized in G1 with α-factor and released at 30ºC.
