# Supplementary material for: Excess of Yra1 RNA-Binding Factor Causes Transcription-Dependent Genome Instability, Replication Impairment and Telomere Shortening
Source: PLoS Genet. 2016 Apr 1;12(4):e1005966. doi: 10.1371/journal.pgen.1005966 (PMC4818039; doi:10.1371/journal.pgen.1005966)
Supplement: S11 Fig — A representation of each chromosome with the signal log2 ratio values for the significant Rrm3 binding clusters is plotted. The X-axis shows chromosomal coordinates in kb. Centromeres are indicated as open circles. (PDF) [file pgen.1005966.s011.pdf]

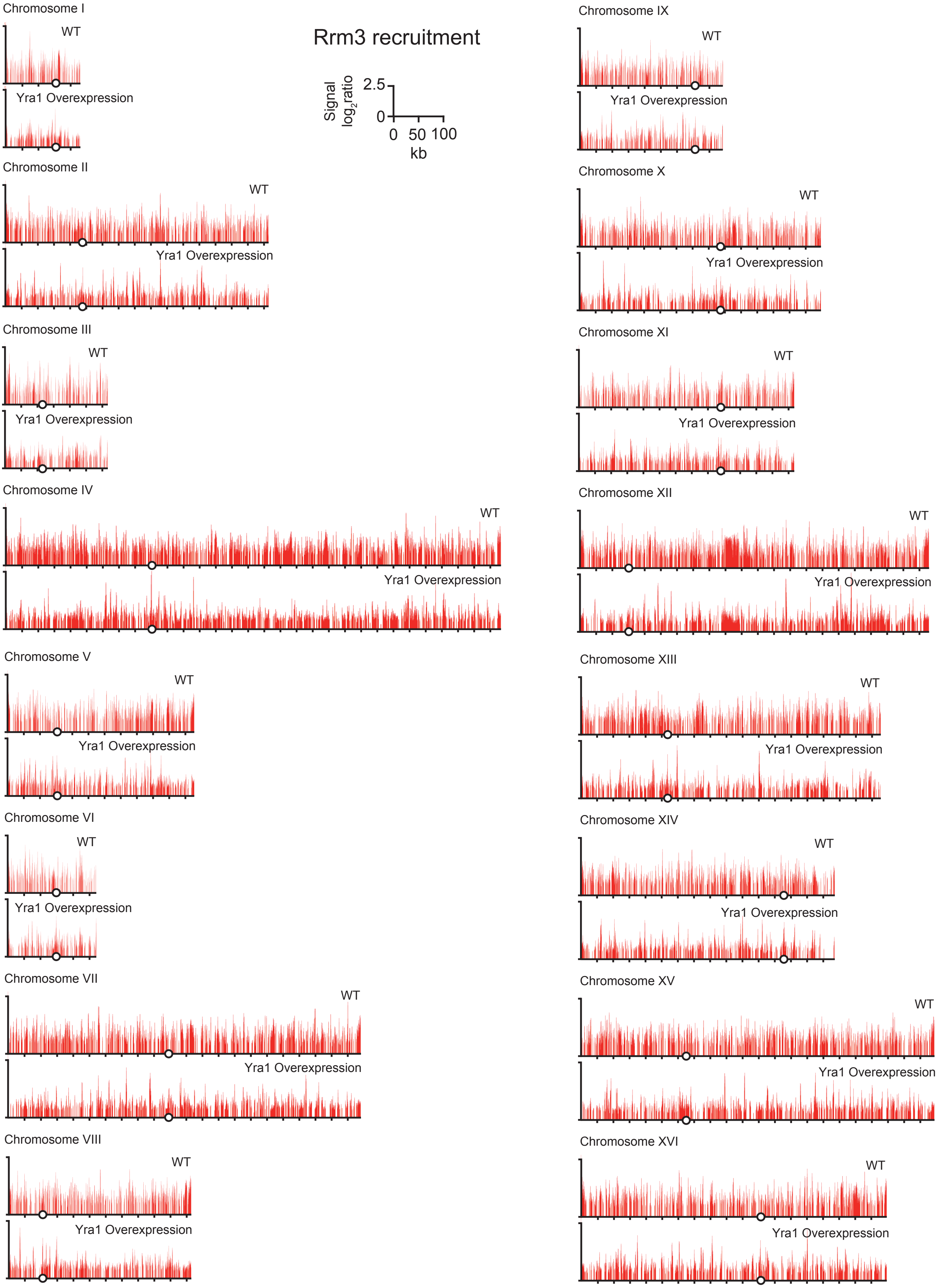

S11 Figure. Genomic view of Rrm3 recruitment under wild-type (*GAL::YRA1*) and Overexpression (*GAL::YRA1Δi*) conditions. A representation of each chromosome with the signal log_2 ratio values for the significant Rrm3 binding clusters is plotted. The X-axis shows chromosomal coordinates in kb. Centromeres are indicated as open circles.
