# Supplementary material for: Excess of Yra1 RNA-Binding Factor Causes Transcription-Dependent Genome Instability, Replication Impairment and Telomere Shortening
Source: PLoS Genet. 2016 Apr 1;12(4):e1005966. doi: 10.1371/journal.pgen.1005966 (PMC4818039; doi:10.1371/journal.pgen.1005966)
Supplement: S12 Fig — (A) Venn diagrams showing the overlap between genes with significant Rrm3 binding in the different ChIP-chip experiments. (B) Venn diagrams showing the overlap between genes with significant Yra1 and Rrm3 binding in different ChIP-chip experiments. (C) Rrm3 cluster distribution at ARSs, centromeres, introns, ncRNA, transponible elements, RNAPIII genes, snoRNA/snRNA and telomeres under wild-type (GAL::YRA1) and Overexpression (GAL::YRA1Δi) conditions. (PDF) [file pgen.1005966.s012.pdf]

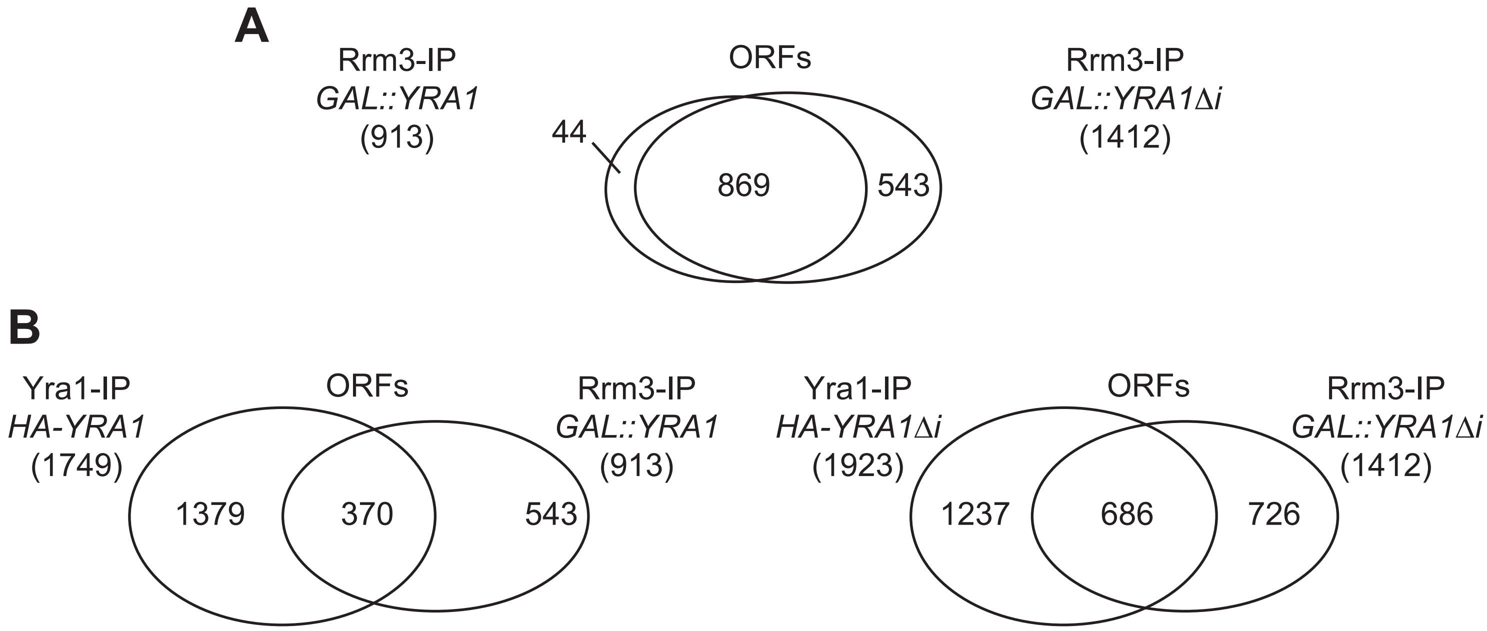

C

Rrm3-IP Recruitment

	GAL::YRA1	*GAL::YRA1Δi*	Total
ARS	33	34	336
Centromeres	3	1	16
Introns	35	44	376
ncRNA	4	4	15
Retrotransposon	46	48	50
Transposable element	83	85	89
rRNA	17	24	27
snoRNA/snRNA	6	5	83
tRNA	18	28	299
Telomeres	26	25	63
X' Element	58	58	60
Y' Element	18	19	19

S12 Figure. (A) Venn diagrams showing the overlap between genes with significant Rrm3 binding in the different ChIP-chip experiments. **(B)** Venn diagrams showing the overlap between genes with significant Yra1 and Rrm3 binding in different ChIP-chip experiments. **(C)** Rrm3 cluster distribution at ARSs, centromeres, introns, ncRNA, transponible elements, RNAPIII genes, snoRNA/snRNA and telomeres under wild-type (*GAL::YRA1*) and Overexpression (*GAL::YRA1Δi*) conditions.
