# Supplementary material for: Excess of Yra1 RNA-Binding Factor Causes Transcription-Dependent Genome Instability, Replication Impairment and Telomere Shortening
Source: PLoS Genet. 2016 Apr 1;12(4):e1005966. doi: 10.1371/journal.pgen.1005966 (PMC4818039; doi:10.1371/journal.pgen.1005966)
Supplement: S1 Table — Top genes whose expression levels are up-regulated and down-regulated in YRA1-overexpressing cells (GAL::YRA1Δi). (PDF) [file pgen.1005966.s013.pdf]

S1 Table - Genes up- and down-regulated in *YRA1*-overexpressing cells

Up-regulated	Gene name	Fold-change
YAL065C		1,578435209
YAR073W	IMD1	1,459980842
YBL051C	PIN4	1,23556803
YBL075C	SSA3	3,941014096
YBL078C	ATG8	1,345717144
YBL084C	CDC27	1,257160297
YBR021W	FUR4	1,458655738
YBR072W	HSP26	3,514191943
YBR101C	FES1	1,205077236
YBR212W	NGR1	1,305521577
YBR219C		2,147760534
YBR284W		2,448945447
YBR298C-A		2,766435286
YBR301W	PAU24	2,60743117
YCR104W	PAU3	2,015599349
YDL020C	RPN4	1,655652589
YDL101C	DUN1	1,273123522
YDL148C	NOP14	1,213605232
YDL159W	STE7	1,236264803
YDL168W	SFA1	1,269977075
YDL243C	AAD4	1,572776814
YDL243C	AAD4	1,572776814
YDR027C	VPS54	1,311632155
YDR034C-A		1,316834534
YDR042C		1,995260777
YDR054C	CDC34	1,218281749
YDR082W	STN1	1,208704553
YDR146C	SWI5	1,345733216
YDR171W	HSP42	1,583744277
YDR173C	ARG82	1,329642942
YDR182W-A		1,973174585
YDR195W	REF2	1,253686039
YDR207C	UME6	1,213317613
YDR218C	SPR28	2,070083917
YDR228C	PCF11	1,265773811
YDR246W-A		1,660363817
YDR256C	CTA1	1,552289033
YDR259C	YAP6	1,533181626
YDR273W	DON1	1,39027791
YDR381W	YRA1	2,295770626
YDR386W	MUS81	1,393126378
YDR402C	DIT2	1,638890844
YDR446W	ECM11	2,01141839
YDR501W	PLM2	1,416973872
YDR522C	SPS2	1,544438009
YDR523C	SPS1	2,265912181
YEL023C		1,355512312
YEL069C	HXT13	2,128119871
YEL069C	HXT13	2,128119871

Down-regulated	Gene name	Fold-change
YBR022W	CNE1	2,192734343
YBR033W		1,549425411
YDL079C		1,995328085
YDL104C	POA1	1,602812917
YDR031W	EDS1	1,681420248
YDR352W		1,601293482
YDR536W		2,394386569
YEL057C		1,849992835
YER018C		1,530840918
YGL007C-A		1,600752795
YGR041W		1,772404792
YGR052W	NPP1	1,54060004
YGR249W	ERP3	2,711472488
YHL012W	PUS9	1,931010433
YJL157C	MRK1	1,543886068
YJR131W	PMT1	1,532737646
YJR147W	QRI7	1,728979118
YLR040C	COS7	1,773294957
YLR231C	MIC14	1,500839968
YMR316W	ENA5	1,577371746
YMR319C	RRG1	1,603049897
YNL237W	IPT1	1,578220418
YNL259C	TMN2	1,716408551
YNR067C	FIN1	1,722433199
YOR034C-A	GTB1	1,51085649
YOR071C		1,510084483
YOR313C	DAD4	1,59711223
YPL014W		2,065046715
YPL040C		1,502009699

YEL070W	DSF1	1,716610964
YER004W	FMP52	1,385595536
YER021W	RPN3	1,241063759
YER060W-A	FCY22	1,384573761
YER078W-A		1,616274216
YER096W	SHC1	1,932587938
YER103W	SSA4	2,208849348
YER106W	MAM1	2,32836134
YER173W	RAD24	1,409221147
YER179W	DMC1	1,465579752
YFL014W	HSP12	1,221975279
YFL033C	RIM15	1,421222297
YFL053W	DAK2	1,84896569
YFL056C	AAD6	3,877264063
YFR010W	UBP6	1,286107439
YFR027W	ECO1	1,438652261
YFR032C	RRT5	4,665553667
YFR050C	PRE4	1,216988175
YFR057W		2,236420654
YGL062W	PYC1	1,404961916
YGL073W	HSF1	1,234234475
YGL116W	CDC20	1,215873602
YGL122C	NAB2	1,30589786
YGL163C	RAD54	1,31790056
YGL175C	SAE2	1,504650658
YGL176C		1,2513952
YGL244W	RTF1	1,254550668
YGR002C	SWC4	1,212810758
YGR035C		4,92461551
YGR048W	UFD1	1,467574776
YGR059W	SPR3	1,402212173
YGR142W	BTN2	2,590458726
YGR146C-A		2,08999086
YGR180C	RNR4	1,250125491
YGR211W	ZPR1	1,282257369
YGR223C	HSV2	1,284843997
YGR238C	KEL2	1,280349965
YGR286C	BIO2	1,48924861
YHL016C	DUR3	1,65650195
YHR007C-A		3,523748982
YHR014W	SPO13	1,74240177
YHR021W-A	ECM12	1,805067009
YHR027C	RPN1	1,311531867
YHR075C	PPE1	1,195033629
YHR079C-A	SAE3	3,017197723
YHR086W-A		1,331873949
YHR157W	REC104	1,625128364
YHR179W	OYE2	1,452933804
YHR185C	PFS1	1,255210996
YHR216W	IMD2	1,372140463
YIL038C	NOT3	1,193594375
YIL107C	PFK26	1,382672332
YIL143C	SSL2	1,612736964

YIL144W	TID3	1,285953873
YIR013C	GAT4	2,326727444
YJL110C	GZF3	1,28640183
YJL218W		1,294918093
YJL219W	HXT9	1,4505375
YJL219W	HXT9	1,4505375
YJR005C-A		4,716371824
YJR010W	MET3	1,497305677
YJR047C	ANB1	1,317594077
YJR120W		1,777045244
YKL043W	PHD1	1,381858124
YKL070W		3,079053451
YKL106C-A		1,577013025
YKL125W	RRN3	1,595309567
YKL221W	MCH2	1,62327449
YKR071C	DRE2	1,230066549
YKR093W	PTR2	1,342653502
YLL046C	RNP1	4,431333065
YLL060C	GTT2	1,724233613
YLL064C	PAU18	1,570319742
YLL066W-B		1,272501939
YLR012C		2,771255448
YLR109W	AHP1	1,453344474
YLR134W	PDC5	1,328941218
YLR137W	RKM5	1,387764011
YLR216C	CPR6	1,34050033
YLR225C		1,296285889
YLR329W	REC102	1,811832488
YLR343W	GAS2	1,582433131
YLR362W	STE11	1,30378304
YLR363C	NMD4	1,306679674
YLR376C	PSY3	1,364118101
YLR406C-A		1,475927985
YLR424W	SPP382	1,22700485
YLR431C	ATG23	1,36681754
YLR433C	CNA1	1,287146427
YLR460C		3,662355029
YLR461W	PAU4	2,658244684
YML007W	YAP1	1,57652901
YML032C	RAD52	1,336978538
YML043C	RRN11	1,304361484
YML058W-A	HUG1	5,109115552
YML100W-A		1,281395482
YML112W	CTK3	1,183120151
YMR004W	MVP1	1,204450417
YMR065W	KAR5	1,273756958
YMR067C	UBX4	1,296766234
YMR175W	SIP18	3,147689806
YMR177W	MMT1	1,502935715
YMR180C	CTL1	1,25893974
YMR191W	SPG5	1,367817774
YMR192W	GYL1	1,259295248
YMR242W-A		1,614890948

YMR276W	DSK2	1,233497011
YNL036W	NCE103	1,546282657
YNL064C	YDJ1	1,224556992
YNL130C-A	DGR1	1,409548268
YNL155W		1,489073652
YNL251C	NRD1	1,570907974
YNR062C		1,574928499
YOL017W	ESC8	1,304622366
YOL019W-A		1,389421109
YOL024W		1,557217077
YOL045W	PSK2	1,350289268
YOL047C		1,549745653
YOL123W	HRP1	1,555998535
YOL149W	DCP1	1,594326141
YOL151W	GRE2	2,338653446
YOL156W	HXT11	1,728760358
YOL162W		1,749575379
YOR008C-A		1,250658534
YOR009W	TIR4	1,431386551
YOR023C	AHC1	1,547619656
YOR027W	STI1	1,275107079
YOR068C	VAM10	1,678161706
YOR076C	SKI7	1,275368874
YOR161C-C		2,424216161
YOR177C	MPC54	1,260820574
YOR183W	FYV12	1,760073686
YOR186W		1,891052844
YOR214C		2,809221299
YOR237W	HES1	2,875337587
YOR242C	SSP2	2,573960153
YOR357C	SNX3	1,197915415
YOR387C		1,34367537
YOR394W	PAU21	1,725633241
YPL021W	ECM23	1,65648015
YPL027W	SMA1	2,191932184
YPL041C		1,631172814
YPL051W	ARL3	1,396650058
YPL054W	LEE1	1,494397907
YPL130W	SPO19	6,040213285
YPL153C	RAD53	1,282117844
YPL164C	MLH3	1,502553874
YPL169C	MEX67	1,227746924
YPL171C	OYE3	3,543445069
YPL192C	PRM3	1,300000302
YPL240C	HSP82	1,370412039
YPL245W		1,479561904
YPL255W	BBP1	1,238011804
YPR025C	CCL1	1,252478415
YPR158W	CUR1	1,515126588
YPR192W	AQY1	1,512225924
