# Supplementary material for: Excess of Yra1 RNA-Binding Factor Causes Transcription-Dependent Genome Instability, Replication Impairment and Telomere Shortening
Source: PLoS Genet. 2016 Apr 1;12(4):e1005966. doi: 10.1371/journal.pgen.1005966 (PMC4818039; doi:10.1371/journal.pgen.1005966)
Supplement: S2 Table — (PDF) [file pgen.1005966.s014.pdf]

Table S2. Yeast strains used in this study.

Source	Genotype	Source
AYW3-1B	*MATa ade2 can1-100 his3 leu2-3,112 leu2-k::ADE2-URA3::leu2-k trp1-1 ura3-1 rad5-G35R*	[1]
A3Y3A	*MATα ura3 ade2 his3leu2k::URA3-ADE2::leu2k*	[2]
BY4741	*MATa his3Δ1 leu2Δ0 met15Δ ura3Δ0*	Euroscarf
Y06401	BY4741 *rad51Δ::KanMX*	Euroscarf
Y00540	BY4741 *rad52Δ::KanMX*	Euroscarf
Y03576	BY4741 *rad9Δ::KanMX*	Euroscarf
Y04222	BY4741 *mus81Δ::KanMX*	Euroscarf
Y00775	BY4741 *sgs1Δ::KanMX*	Euroscarf
Y01331	BY4741 *srs2Δ::KanMX*	Euroscarf
W303-1A	*MATa ade2-1 can1-100 his3-11,15 leu2-3,112 trp1-1 ura3-1 rad5-G535R*	R. Rothstein
	W303-1A *sml1Δ::URA3*	J.A. Tercero
YJT75	W303-1A *sml1Δ::URA3 rad53Δ::LEU2*	J.A. Tercero
DD303	W303-1A *elg1Δ::KanMX*	[3]
DD377	W303-1A *ctf18Δ::KanMX*	[3]
DD379	W303-1A *rad24Δ::KanMX*	[3]
YLL134	W303-1A *mec3Δ::TRP1*	[4]
DKY194	W303-1A *dia2Δ::KanMX*	[5]
AMH4	*MAT α pol30-52 ade3::GAL::HO)*	[6]
WRBb-9B	*MATa ade2-1 bar1Δ can1-100 his3-11,15 leu2-3,112 RRM3::FLAG::KanMX ura3::URA3/GPD-Tk(7x)*	[7]
YRA1 shuffle	*MATa,ade2,his3,leu2,trp1,ura3,yra1::HIS3 (pRS316-YRA1)*	[8]
yra1-1	*MATa,ade2,his3,leu2,trp1,ura3,yra1::HIS3 (pRS314-yra1-1)*	[8]
YRA1Δi	*MATa,ade2,his3,leu2,trp1,ura3,yra1::HIS3 (pHA-YRA1Δi)*	[9]
YRA1ΔRBDΔi	*MATa,ade2,his3,leu2,trp1,ura3,yra1::HIS3 (pHA-YRA1ΔRBD)*	[9]
U678-4C	W303-1B *hpr1Δ::HIS3*	[10]

1. Piruat JI, Aguilera A (1996) Mutations in the yeast SRB2 general transcription factor suppress hpr1-induced recombination and show defects in DNA repair. Genetics 143: 1533-1542.
2. Aguilera A, Klein HL (1990) HPR1, a novel yeast gene that prevents intrachromosomal excision recombination, shows carboxy-terminal homology to the Saccharomyces cerevisiae TOP1 gene. Mol Cell Biol 10: 1439-1451.
3. Kanellis P, Agyei R, Durocher D (2003) Elg1 forms an alternative PCNA-interacting RFC complex required to maintain genome stability. Curr Biol 13: 1583-1595.
4. Giannattasio M, Sommariva E, Vercillo R, Lippi-Boncambi F, Liberi G, et al. (2002) A dominant-negative MEC3 mutant uncovers new functions for the Rad17 complex and Tel1. Proc Natl Acad Sci U S A 99: 12997-13002.
5. Swaminathan S, Kile AC, MacDonald EM, Koepp DM (2007) Yra1 is required for S phase entry and affects Dia2 binding to replication origins. Mol Cell Biol 27: 4674-4684.

6. Holmes AM, Haber JE (1999) Double-strand break repair in yeast requires both leading and lagging strand DNA polymerases. Cell 96: 415-424.
7. Santos-Pereira JM, Herrero AB, Garcia-Rubio ML, Marin A, Moreno S, et al. (2013) The Npl3 hnRNP prevents R-loop-mediated transcription-replication conflicts and genome instability. Genes Dev 27: 2445-2458.
8. Strasser K, Hurt E (2000) Yra1p, a conserved nuclear RNA-binding protein, interacts directly with Mex67p and is required for mRNA export. Embo J 19: 410-420.
9. Zenklusen D, Vinciguerra P, Strahm Y, Stutz F (2001) The yeast hnRNP-Like proteins Yra1p and Yra2p participate in mRNA export through interaction with Mex67p. Mol Cell Biol 21: 4219-4232.
10. Piruat JI, Aguilera A (1998) A novel yeast gene, THO2, is involved in RNA pol II transcription and provides new evidence for transcriptional elongation-associated recombination. EMBO J 17: 4859-4872.
