# Supplementary material for: Excess of Yra1 RNA-Binding Factor Causes Transcription-Dependent Genome Instability, Replication Impairment and Telomere Shortening
Source: PLoS Genet. 2016 Apr 1;12(4):e1005966. doi: 10.1371/journal.pgen.1005966 (PMC4818039; doi:10.1371/journal.pgen.1005966)
Supplement: S3 Table — (PDF) [file pgen.1005966.s015.pdf]

Table S3. Primers used in this study.

**Primers**	**Sequence (5'-3')**
sYRA1up	GAAGTAATAGAGCCCGTGTC
sYRA1low	AAACCTTCGACGTTGACCTT
YRAclon up	ATCATTATGGATCCGACCTACATCTGCTAAATGTC
YRAclon low	CACAACGACTGCAGTTAAATCAAACAAAAAATTG
rDNA up	TTGGAGAGGGCAACTTTGG
rDNA low	CAGGATCGGTCGATTGTGC
Telom up	TGCCGTGCAACAAACACTAAATCAA
Telom low	CGCTCGAGAAAGTTGGAGTTTTTCA
TLC1up	TAAGGTGACAGAAAAAAAGG
TLC1low	AACCATCTTGAAAAATCTCA
**qPCR primers**	**Sequence (5'-3')**
18S F	CGATCCCTAGTCGGCATAGT
18S R	GAGGTGAAATTCTTGGATTTATTG
PMA1 F	ATCGCTATTTTCGCTGATGTTG
PMA1 R	CGGGCTTTGGAGAGTAAGGA
ts(AGA)J F	AGTGGTTAAGGCGAAAGATTAGAAAT
ts(AGA)J R	GACAACTGCAGGACTCGAACCT
IME1 F	GATATGCTGCGGCTTACTCCTT
IME1 R	ATTTCTTGAAACCTGACCTTGTCAT
ARS1211-1 S	GTTTCCTCCACCTCCTTTGTGT
ARS1211-1 AS	TGACCGATATATTGTGTTTCTATACTGTGT
ARS1211-2 S	CGTTCAATTCGTTGGCGTTAC
ARS1211-2 AS	TTAACACCGTTTTCGGTTTGC
ARS508-1 S	CCCGTGGTAAACCTTTAGAAAAAC
ARS508-1 AS	ATATGAACGGCAAATTGAGACAAA
ARS508-2 S	AGTCATTAATAGCAAAGCCGTACGT
ARS508-2 AS	GGTCCTTTGATGTAACGATCATATTG
