# Supplementary material for: Excess of Yra1 RNA-Binding Factor Causes Transcription-Dependent Genome Instability, Replication Impairment and Telomere Shortening
Source: PLoS Genet. 2016 Apr 1;12(4):e1005966. doi: 10.1371/journal.pgen.1005966 (PMC4818039; doi:10.1371/journal.pgen.1005966)
Supplement: S10 Fig — Other details as in Fig 8. (PDF) [file pgen.1005966.s010.pdf]

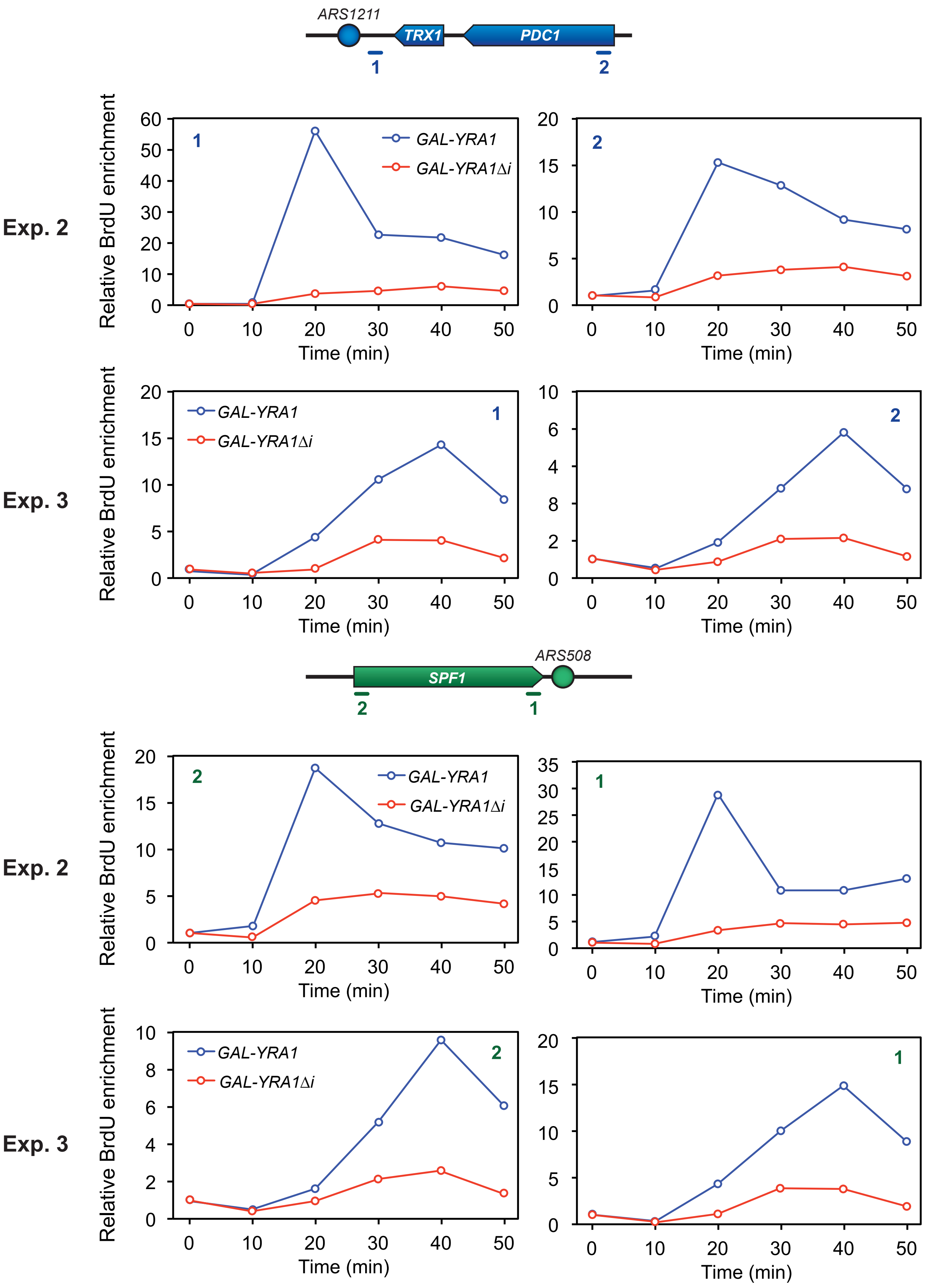

S10 Figure. BrdU incorporation analysis for two independent experiments in WRBb-9B (WT) cells overexpressing (*GAL-YRAΔi*) or not (*GAL-YRA1*) *YRA1*. Other details as in Figure 7.
